# Glucose controls manganese homeostasis through transcription factors regulating known and newly identified manganese transporter genes in *Bacillus subtilis*

**DOI:** 10.1016/j.jbc.2023.105069

**Published:** 2023-07-17

**Authors:** Mitsuo Ogura, Minenosuke Matsutani, Kei Asai, Michio Suzuki

**Affiliations:** 1 Institute of Oceanic Research and Development, Tokai University, Shizuoka, Japan; 2 NODAI Genome Research Center, Tokyo University of Agriculture, Tokyo, Japan; 3 Department of Bioscience, Tokyo University of Agriculture, Tokyo, Japan; 4 Graduate School of Agricultural and Life Sciences, The University of Tokyo, Tokyo, Japan

**Keywords:** manganese homeostasis, manganese transporter genes, glucose induction, RNA-Seq, metabolic homeostasis

## Abstract

Mn^2+^ is an essential nutrient whose concentration is tightly controlled in bacteria. In *Bacillus subtilis*, the Mn^2+^-activated transcription factor MntR controls Mn^2+^ transporter genes. However, factors regulating intracellular Mn^2+^ concentration are incompletely understood. Here, we found that glucose addition induces an increase in intracellular Mn^2+^ concentration. We determined this upshift was mediated by glucose induction of the major Mn^2+^ importer gene *mntH* by the transcription factor AhrC, which is known to be involved in arginine metabolism and to be indirectly induced by glucose. In addition, we identified novel AhrC-regulated genes encoding the Mn^2+^ importer YcsG and the ABC-type exporter YknUV. We found the expression of these genes was also regulated by glucose and contributes to the glucose induction of Mn^2+^ concentrations. *ycsG* expression is regulated by MntR as well. Furthermore, we analyzed the interaction of AhrC and MntR with the promoter driving *ycsG* expression and examined the Mn^2+^-dependent induction of this promoter to identify the transcription factors responsible for the Mn^2+^ induction. RNA-Seq revealed that disruption of *ahrC* and *mntR* affected the expression of 502 and 478 genes, respectively (false discovery rate, <0.001, log_2_[fold change] ≥ |2|. The AhrC- and/or MntR-dependent expression of twenty promoters was confirmed by LacZ analysis, and AhrC or MntR binding to some of these promoters was observed *via* EMSA. The finding that glucose promotes an increase in intracellular Mn^2+^ levels without changes in extracellular Mn^2+^ concentrations is reasonable for the bacterium, as intracellular Mn^2+^ is required for enzymes and pathways mediating glucose metabolism.

Glucose is the most favored carbon source for many bacteria; therefore, bacteria have developed several glucose-responsive systems (1, 2). In Gram-positive bacteria, including *Bacillus subtilis*, catabolite control protein A (CcpA) is the master transcription regulator for carbon catabolite regulation (1, 2). Incorporating glucose into bacterial cells increases the levels of metabolites such as fructose-1,6-bisphosphate in the glycolysis pathway, which triggers HPr phosphorylation at Ser46. HPr is a phosphocarrier protein in the sugar phosphotransferase system, and P-Ser-HPr activates CcpA, resulting in large transcriptome changes. Moreover, there are several additional glucose-responsive transcription factors, such as CcpC, CcpN, CggR, and GlcT (2). CcpN regulates the structural genes involved in gluconeogenesis (3). Our recent studies also revealed a glucose-responsive system that includes the nucleoid-associated protein YlxR (4, 5, 6) and *ywlE* encoding a phosphatase for protein Arg-phosphate, which is a regulatory factor for *ylxR* expression (7). Glucose induced *ywlE* expression through an unknown mechanism, leading to the induction of *ylxR* expression (7). YwlE counteracts arginine phosphorylation of proteins by McsB kinase, thus protecting the protein from degradation (8). Proteomic analyses have revealed that most glycolytic enzymes are targets of McsB (8), which suggests that YwlE protects glycolytic enzymes from degradation (7).

Mn^2+^ is an essential nutrient in organisms, including bacteria such as *B. subtilis*, because it plays roles in cell differentiation, biofilm formation, many metabolic pathways, and stress responses including oxidative stress (9, 10, 11, 12, 13, 14, 15, 16, 17). In *Streptococci*, the intracellular Mn^2+^ concentration affects the glycolytic enzymes expression and metabolome (10, 11). In *Staphylococcus aureus*, glycolysis increases the cellular demand for Mn^2+^ and this bacterium has a metal-independent and a metal (Mn^2+^ or Zn^2+^)-dependent fructose 1,6-bisphosphate aldolase, which enables *S. aureus* to resist against host-imposed Mn^2+^ limitation (12). In *Bacillus*, Mn^2+^ is a cofactor for two glycolytic enzymes, namely phosphoglycerate mutase and pyruvate kinase (18, 19), indicating a link between glycolytic enzymes and Mn^2+^. However, excess Mn^2+^ is harmful and causes intoxication, mainly through mismetallation of proteins (16, 17). Thus, intracellular Mn^2+^ concentrations are tightly regulated at transcription and post-transcription levels (20, 21). In *B. subtilis*, the Mn^2+^-activated transcription factor MntR plays a central role in Mn^2+^ homeostasis (21). *B. subtilis* is known to contain two Mn^2+^ importers, MntABCD (ABC-type transporter) and MntH (primary importer), and major and minor exporters MneP and MneS, respectively, both of which are cation diffusion facilitators (22, 23). TerC-type transporters YkoY and YceF have also been reported; however, their involvement in Mn^2+^ transport remains unclear (23). When intracellular Mn^2+^ concentrations are low, MntR does not repress importer genes or activate exporter genes. In contrast, MntR represses importer genes under high intracellular Mn^2+^ concentrations, and a further upshift in Mn^2+^ concentrations leads to MntR-mediated activation of exporter genes. This MntR-mediated Mn^2+^ homeostasis is dependent on the fact that MntR requires higher levels of Mn^2+^ to activate exporter genes (24). In other bacteria, for example *Escherichia coli*, Mn^2+^ homeostasis is maintained by MntR and additional transcription factors such as H-NS, OxyR, and ferric uptake regulator (17).

We started this study from the mechanistic analysis of the previous observation of glucose induction (GI) of *ywlE*. We found GI of Mn^2+^ concentrations, leading to GI of *ywlE* expression. The GI of translation of AhrC, an arginine metabolism-controlling transcription factor, is already known (25, 26, 27), and we found that AhrC regulates the four known MntR-regulated Mn^2+^ transporter loci. RNA-Seq analysis of *ahrC* and *mntR* disruptants resulted in the identification of two new Mn^2+^ transporter loci, contributing to the GI of Mn^2+^ concentrations.

## Results

### Cellular Mn^2+^ concentrations regulate *ywlE* expression

**Figure 1:**
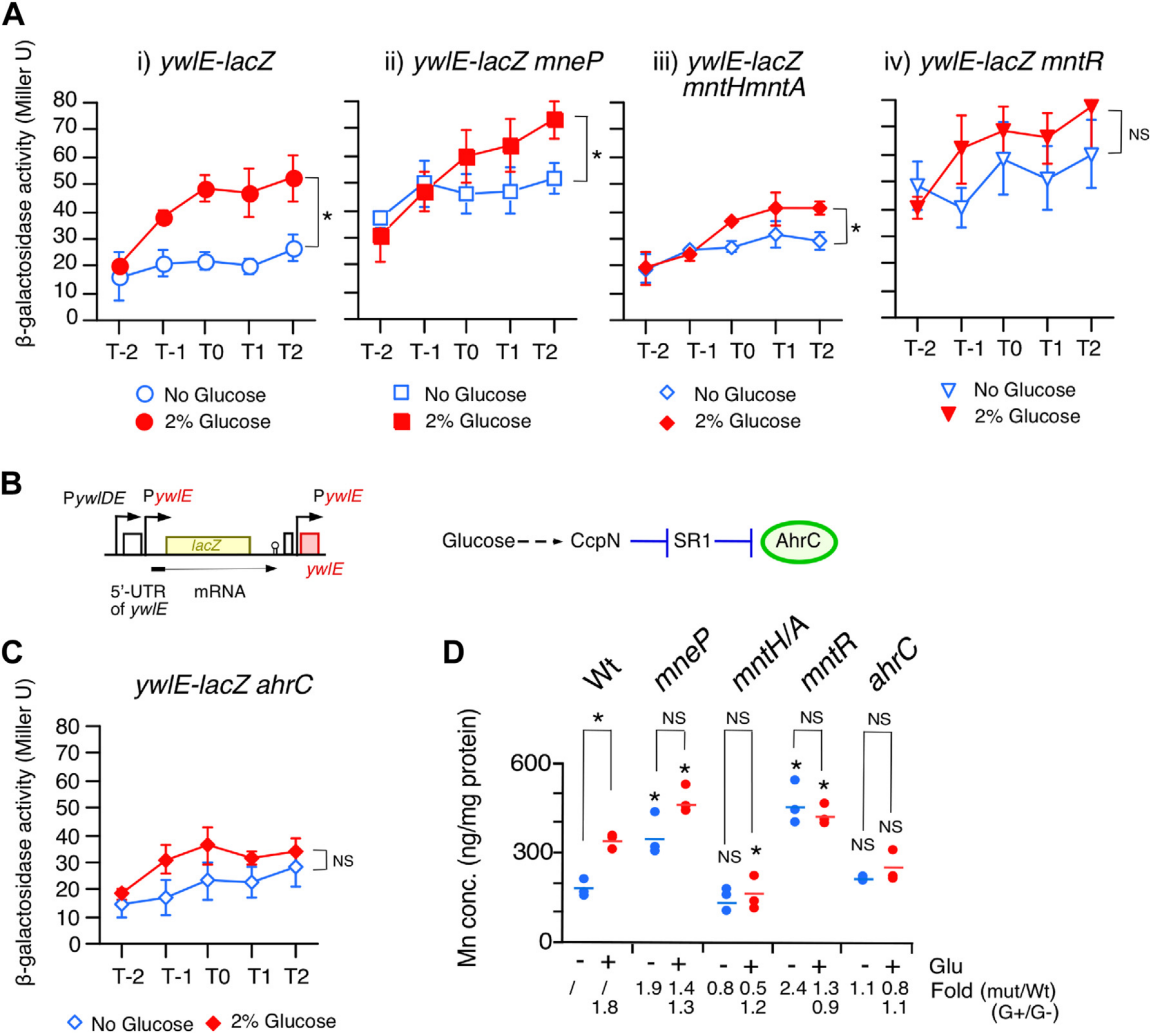
**Glucose induction of *ywlE-lacZ* and increase in cellular Mn concentrations.** (*A*) and (*C*) β-Galactosidase activities were measured by using highly sensitive substrate CPRG shown in Miller units. Means from three independent experiments and the SDs are shown. Significant differences in the effects of glucose addition at T2 were determined using nonpaired *t* test. ∗*p* < 0.05; NS, no significant differences. The *x*-axis represents the growth time in hours relative to the end of vegetative growth (T0). Cells were grown in sporulation medium with (*closed symbols*) or without (*open symbols*) 2% glucose and sampled hourly. Strains: WT (OAM888), and derivatives of OAM888, *mneP* (OAM1004), *mntH/mntA* (OAM1003), *mntR* (OAM1007), and *ahrC* (OAM1006). (*B*) *left*: relevant structure of OAM888. *Box*, *bent arrows*, and *stem–loop* show ORF, promoter, and terminator, respectively. *Right*: flow of glucose-mediated regulation of *ahrC*. *Dotted arrow* and *T-bar* mean indirect activation and direct inhibition, respectively. SR1, small regulatory RNA, inhibits translation of *ahrC*. (*D*) cellular Mn concentrations. T2 cells grown in sporulation medium were harvested and processed. Strains; WT (168), *mneP* (OAM993), *mntH/mntA* (OAM992), *mntR* (OAM996), and *ahrC* (OAM995). “Glu” represents glucose. Three biologically independent samples were measured. Significant differences between Wt and mutants, with or without glucose (∗ and “NS” above each data point indicate *p* < 0.05 and no significant difference, respectively) and the effect of glucose addition to each strain were determined using nonpaired *t* test. ∗*p* < 0.05; NS, no significant differences. The *short horizontal lines* indicate the mean of the data points. CPRG, chlorophenolred β-D-galactopyranoside.

Previously, we observed that *ywlE-lacZ* expression (Fig. 1*B*) was induced by glucose in sporulation medium (i, Fig. 1*A*), which was confirmed at the protein level (7). The *ywlD* gene, whose product is similar to the Mn^2+^ exporter MntP in *E. coli*, is located upstream of *ywlE* (Fig. S1*A*) (28). We observed that glucose completely inhibited P*ywlDE*-*lacZ* at the *amyE* locus (left, Fig. S1*B*), which seems to be incompatible with GI of *ywlE*. We therefore investigated the glucose effect on the overall expression of *ywlE* using (P*ywlDE* plus P*ywlE*)-*lacZ* (Fig. S1*A*). We confirmed GI of *ywlE* using this fusion (middle, Fig. S1*B*). Generally, genes whose function is related to each other in cellular physiology tend to form a cluster in the bacterial genome (29). Thus, we hypothesized that the putative Mn^2+^ exporter is downregulated by glucose, resulting in an increase in cellular Mn^2+^ concentration, somehow leading to the upregulation of *ywlE-lacZ*. Indeed, glucose addition elevated the Mn^2+^ concentration by 1.8-fold, which has not been previously reported, to the best of our knowledge (Fig. 1*D*). To investigate whether YwlD is involved in Mn^2+^ transport, we examined Mn^2+^ concentration in the *ywlD* disruptant and observed no changes of cellular Mn^2+^ concentration and GI of *ywlE-lacZ* (right, Fig. S1, *B* and *C*). We conclude that YwlD is not involved in Mn^2+^ homeostasis.

When the gene encoding the Mn^2+^ exporter MneP was disrupted, Mn^2+^ concentration was increased as expected (Fig. 1*D*), and *ywlE-lacZ* expression was also increased independent of glucose (ii, Fig. 1*A*). Conversely, in the *mntA/mntH* double disruptant of the importer genes, the Mn^2+^ concentration was decreased in the presence of glucose, compared to that in the WT (Fig. 1*D*). Concomitantly, *ywlE-lacZ* expression decreased in the presence of glucose (iii, Fig. 1*A*). Thus, changes in cellular Mn^2+^ concentration cause the changes of *ywlE* expression. This *mntA/mntH* strain showed slightly longer growth lag in the semisynthetic modified competence (MC) medium containing glucose, suggesting a role of the GI of Mn^2+^ (Fig. S2*A*) (30). Notably, in this double mutant, Mn^2+^ concentrations were observed to sustain growth, suggesting the presence of a third unknown importer; this has been reported previously (22). Based on these results, we concluded that *ywlE-lacZ* expression is an indicator of Mn^2+^ concentration. Furthermore, Mn^2+^ concentration is known to increase in the *mntR* disruptant (1.6-fold enhancement in LB medium, 31), which we also confirmed (Fig. 1*D*). Thus, the 1.8-fold change (FC) induced by glucose addition in the WT strain is significant. In addition, we confirmed that higher *ywlE-lacZ* expression was observed with and without glucose in the *mntR* disruptant (iv, Fig. 1*A*). This supports the idea that Mn^2+^ concentration regulates the expression of *ywlE-lacZ*. To elucidate the GI of Mn^2+^ concentrations, we examined Mn^2+^ concentrations in the *ccpA* disruptant; however, compared to the WT, no change in the GI pattern was observed (Fig. S1*C*). Next, we tested Mn^2+^ concentrations in the *ahrC* disruptant, which encodes a transcriptional regulator for arginine metabolism genes and is activated indirectly by glucose (Fig. 1*B*) (26, 27). In the *ahrC* disruptant, GI of neither Mn^2+^ concentration nor *ywlE-lacZ* expression was observed (Fig. 1, *C* and *D*). Hence, *ahrC* is involved in regulating Mn^2+^ concentrations.

### Regulation of four known Mn^2+^ transporter genes by AhrC

**Figure 2:**
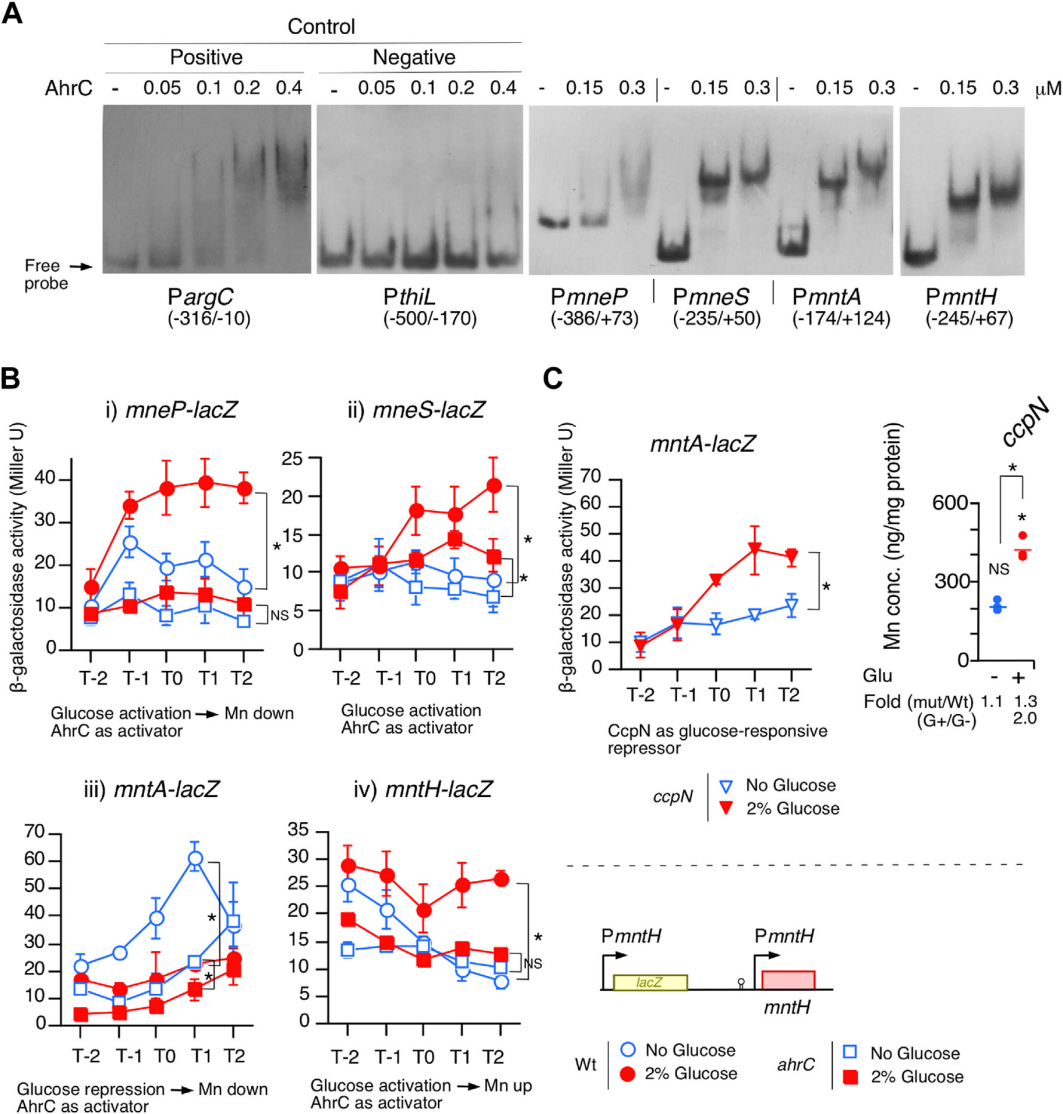
**Involvement of AhrC in Mn**^**2+**^**transporter genes expression by EMSA.** (*A*) EMSA. Protein concentrations and probe names are shown. *Numbers in parentheses* show nucleotides position to the relative to the translation start point for *argC* and *thiL*. For the others, *numbers in parentheses* show nucleotides position to the relative to the transcription start point. (*B*) and *left panel* in (*C*) representsβ-Galactosidase activities. Substrate used was 2-Nitrophenyl-β-D-Galactopyranoside for *mntH-lacZ* and CPRG for the others. Significant differences in the effects of glucose addition at T2 (T1 for *mntA-lacZ*) were determined using nonpaired *t* test. ∗*p* < 0.05; NS, no significant differences. The *x*-axis represents the growth time in hours relative to the end of vegetative growth (T0). Cells were grown in sporulation medium with (*closed symbols*) or without (*open symbols*) 2% glucose and sampled hourly. Strains: P*mneP-lacZ* (wt, OAM1016; *ahrC*, OAM1020), P*mneS-lacZ* (wt, OAM1017; *ahrC*, OAM1021), P*mntA-lacZ* (wt, OAM1014; *ahrC*, OAM1018; *ccpN*, OAM1024). Schematic representation of the structure of the P*mntH-lacZ* fusion is shown. *Box*, *bent arrows*, and *stem–loop* indicate ORF, promoter, and terminator, respectively. *Right panel* in (*C*) shows cellular Mn concentrations in the *ccpN* strain (OAM998). T2 cells grown in sporulation medium were harvested and processed. “Glu” represents glucose. Three biologically independent samples were measured. Significant differences between Wt and mutant, with or without glucose (∗ and “NS” above each data point indicate *p* < 0.05 and no significant difference, respectively) and the effect of glucose addition to the strain were determined using nonpaired *t* test. ∗*p* < 0.05; NS, no significant differences. The *short horizontal lines* indicate the mean of the data points. CPRG, chlorophenolred β-D-galactopyranoside.

In the *ahrC* disruptant, GI of both Mn^2+^ concentration and *ywlE-lacZ* expression were abolished; therefore, we examined whether AhrC regulates Mn^2+^ transporter genes. AhrC was purified and used for the EMSA. Within the protein concentrations at which specific binding was observed (positive control, P*argC*; negative control, P*thiL*, Figure 2*A*), AhrC bound to the promoter regions of all known Mn^2+^ transporter genes (Fig. 2*A*). Next, we constructed transcriptional fusions in the original chromosomal context (Fig. 2*B*) and used them for the expression assay of WT and *ahrC* disruptant with or without glucose. Except for *mntH*, highly sensitive substrate chlorophenolred β-D-galactopyranoside were used for the β-galactosidase assay. We noted that the expression levels of *mntH-lacZ* were at least five times or higher than those of the other fusions when 2-Nitrophenyl-β-D-Galactopyranoside was used as the substrate for all assays (data not shown). For *lacZ* fusion with *mneP* (exporter), glucose activated its expression, leading to a decrease in Mn^2+^ concentration, and further *ahrC* disruption decreased the elevated expression (i, Fig. 2*B*). AhrC functions as an activator irrespective of the presence of glucose. Disruption of *ahrC* abolished the GI of the fusion, indicating that glucose induces fusion through AhrC activation. MneS did not play any role in regulating the Mn^2+^ concentration (Fig. S1*C*), which is consistent with a previous report (23). Glucose also activated *mneS-lacZ*, and *ahrC* functions as an activator, as in the case of *mneP* (ii, Fig. 2*B*). For *lacZ* fusion with *mntA* (importer), glucose repressed its expression, leading to a decrease in Mn^2+^ concentration (iii, Fig. 2*B*). *ahrC* disruption decreased its expression significantly in the absence of glucose, indicating that AhrC functions as an activator. Moreover, glucose-mediated repression in the *ahrC* disruptant was still observed, suggesting no involvement of *ahrC* in glucose repression. We found that *ccpN* disruption resulted in GI of *mntA-lacZ*, suggesting that CcpN is responsible for glucose repression (Fig. 2*C*). These results are consistent with the observation that in the *ccpN* disruptant GI of Mn^2+^ concentration was further enhanced. Since CcpN indirectly activates AhrC, the *ccpN* disruptant should also be considered as an *ahrC*-depleting strain. Thus, for *mntA-lacZ*, the results for the *ccpN* disruptant should be compared to those for the *ahrC* disruptant. For *lacZ* fusion with *mntH* (importer), glucose activated its expression, leading to an increase in Mn^2+^ concentrations, and further *ahrC* disruption decreased elevated expression (iv, Fig. 2*B*). AhrC functions as an activator irrespective of the presence of glucose. Disruption of *ahrC* abolished the GI of the fusion, indicating that glucose induces fusion through AhrC activation. These transporter genes are known to be regulated by MntR (23); thus, these results indicate that all four genes/operons are also regulated by AhrC.

These fusion analyses showed that the effects of glucose and *ahrC* disruption on Mn^2+^ concentrations can be either positive or negative. This may not be consistent with the observation that there was no GI for Mn^2+^ concentrations in the *ahrC* disruptant. Thus, these analyses suggest that there might be AhrC-regulated unknown Mn^2+^ transporter genes.

### Transcriptomes of *ahrC* and *mntR* disruptants

**Figure 3:**
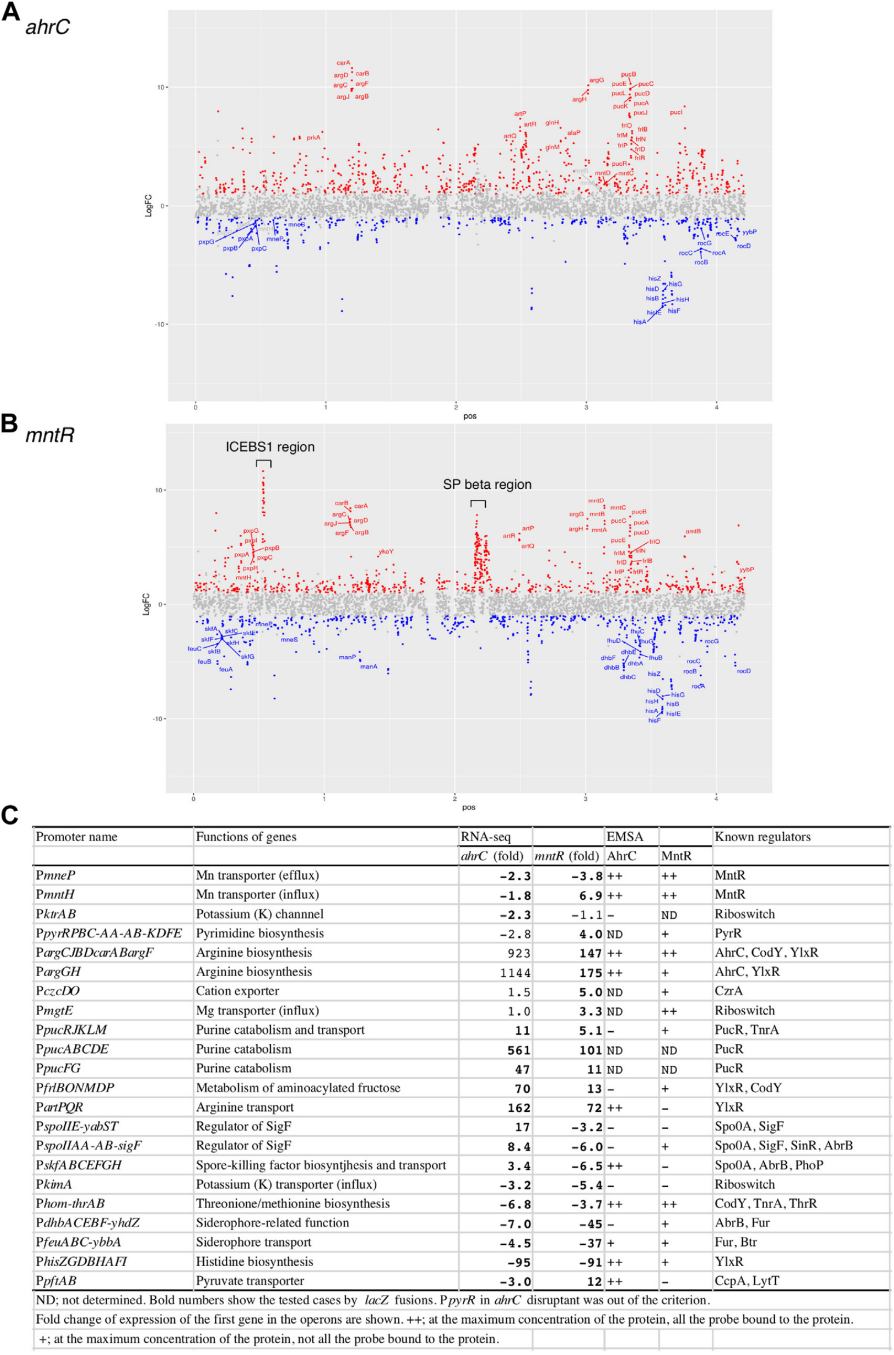
**Comparative RNA-Seq analyses of the *ahrC* and *mntR* mutants.** Values of fold change of transcripts between WT and *ahrC* (*A*) or *mntR* (*B*) mutant cells were calculated from four independent RNA-Seq analyses that were performed using cells at T2 in sporulation medium with 2% glucose. All genes (ordered clockwise from the +1 position of the chromosome) are plotted against fold-change values. *Red* and *blue points* represent upregulation and downregulation, respectively. (*C*) confirmation of the expression of the selected promoters by β-Gal analysis and EMSA. The data or images of both analyses are shown in this figure.

To identify unidentified Mn^2+^ transporter genes, we first determined the transcriptomic profile of the *ahrC* disruptant in the presence of glucose through comparative RNA-Seq analysis of the *ahrC* disruptant. The results using four biological replicates are shown in Figure 3*A* and Table S1. To determine whether new transporter genes are present in the MntR-regulated genes, we performed RNA-Seq analysis of the *mntR* disruptant in the presence of glucose using four biological replicates (Fig. 3*B* and Table S1). In both cases a very large number of AhrC- and/or MntR-regulated genes were observed. The *ahrC* and *mntR* disruptants showed the growth profiles similar to that in the WT strain, excluding secondary effects due to growth retardation (Fig. S2*C*).

We chose 20 operons composed of metabolic and transporter genes, whose expression is affected by *ahrC* and/or *mntR* disruption (Fig. 3*C*), and investigated the reproducibility of expression changes, and confirmed the *mntR*- and/or *ahrC*-dependent regulation of all promoters (Fig. S3*A*). We examined protein binding to these promoter regions using EMSA and identified six new targets for AhrC and twelve new targets for MntR (Fig. S3*B*). These results also suggest that many of the differentially expressed genes (DEGs) in the *ahrC/mntR* disruptants are indirectly regulated by AhrC/MntR.

While AhrC was thought to bind to only eight Arg-box in the operons related to arginine metabolism (32), our analysis revealed 1176 candidate targets (false discovery rate [FDR] <0.05, log_2_[FC] ≥ |1|). Among the DEGs in the *ahrC* disruptant, we detected all eight known operons in addition to histidine and purine metabolism genes (Fig. 3).

We have identified 1316 candidate targets for the MntR regulated genes, including the known directly MntR-regulated loci (FDR <0.05, log_2_[FC] ≥ |1|). Among these, we found large fluctuations in the expression of genes belonging to the SPbeta phage and ICE*Bs*1, which is an integrative and conjugative element (Fig. 3*B*) (33), although their physiological roles in MntR-dependent regulation are currently unknown. The members of the MntR-regulated genes would be affected by intracellular Mn^2+^ concentrations because MntR is activated by Mn^2+^ binding (22). Hence, the expression of more than 1300 genes could potentially be affected by Mn^2+^. Indeed, MntR binding to the newly identified target gene *argC* was affected by Mn^2+^ (Fig. S3*C*).

Interestingly, 472 genes were identified to have altered expression pattern in both *ahrC* and *mntR* disruptants. Enrichment analyses were performed for both transcriptomes (Fig. S4). These results revealed that many metabolic genes including several amino acid biosynthetic genes were observed in both DEGs.

### Identification of candidate AhrC-regulated Mn^2+^ transporter genes

**Figure 4:**
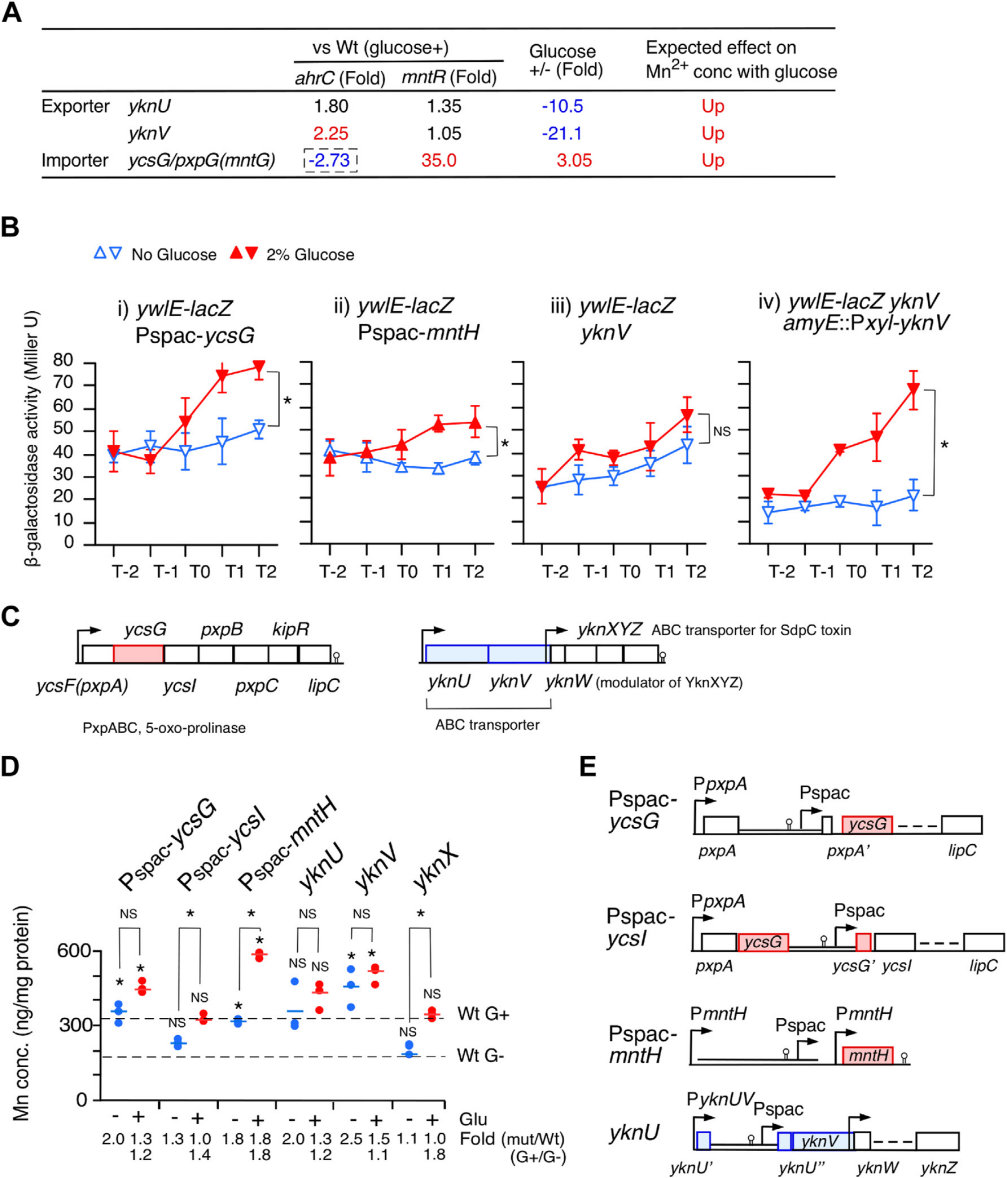
**Newly identified Mn**^**2+**^**transporters.** (*A*) Fold changes of three candidate genes involved in Mn^2+^ transport in RNA-Seq. Fold changes in RNA-Seq when glucose was added to the WT were given (35). The *dotted square* indicates that the decreased expression in the *ahrC* strain using RNA-Seq, while in the β-Gal analysis of the fusion, the increased expression was observed (see Fig. 5*B*). *B*, β-Galactosidase activities. CPRG was used as substrate. Significant differences in the effects of glucose addition at T2 were determined using nonpaired *t* test. ∗*p* < 0.05; NS, no significant differences. The *x*-axis represents the growth time in hours relative to the end of vegetative growth (T0). Cells were grown in sporulation medium with (*closed symbols*) or without (*open symbols*) 2% glucose and sampled hourly. Strains: Pspac-*mntH* (OAM1009), Pspac-*ycsG* (OAM1010), *yknV* (OAM1011), and *yknV amyE*::Px-*yknV* (OAM1012). 0.2 mM and 0.1 mM IPTG was added, 0.2 mM for Pspac-*mntH* and 0.1 mM for Pspac-*ycsG*, respectively. For P*yknU-lacZ* no xylose was added. *C* and *E*, schematic representation of the structure of the *ycsG*-containing operon and *yknUV* operon, and Pspac-*ycsG*, Pspac-*ycsI*, Pspac-*mntH*, and *yknU*. *Box*, *bent arrows*, and *stem–loop* indicate ORF, promoter, and terminator, respectively. *Double lines* indicate the inserted plasmid sequences. *D*, cellular Mn concentrations. T2 cells grown in sporulation medium were harvested and processed. “Glu” represents glucose. Three biologically independent samples were measured. Significant differences between Wt and mutants, with or without glucose (∗ and “NS” above each data point indicate *p* < 0.05 and no significant difference, respectively) and the effect of glucose addition to each strain were determined using nonpaired *t* test. ∗*p* < 0.05; NS, no significant differences. The *short horizontal lines* indicate the mean of the data points. Strains: Pspac-*ycsI* (OAM1002), Pspac-*mntH* (OAM1000), *yknU* (YKNUd), *yknV* (OAM999), and *yknX* (YKNXd). 0.1 mM, 0.5 mM, 0.2 mM, and 1 mM IPTG was added for Pspac-*ycsG*, Pspac-*ycsI*, Pspac-*mntH*, and *yknU*, respectively. CPRG, chlorophenolred β-D-galactopyranoside.

To identify candidate Mn^2+^ transporter genes, DEGs in the *ahrC* disruptant were screened. We searched for disruptants without GI of *ywlE-lacZ* among several AhrC-activated importers with unknown substrates that were under GI or AhrC-repressed exporters with unknown substrates that were under glucose repression (Fig. 4*A*). Next, we examined *ywlE-lacZ* expression in the disruptants. The *ycsG* gene encodes an importer with an unknown substrate, which has been annotated as a 5-oxoproline importer; however, the evidence for this annotation is limited (34), although the other members of this operon are involved in utilizing 5-oxoproline as a carbon source (Fig. 4*C*) (34). In addition to the GI observed in our RNA-Seq (35), the GI of this operon has been previously reported (36). Initial attempts to construct the *ywlE-lacZ* strain with *ycsG* disruption resulted in a highly unstable strain with respect to its no-GI phenotype (data not shown); thus, we adopted an overproduction strategy. Pspac is an IPTG-inducible promoter and Pspac-*ycsG* elevated *ywlE-lacZ* expression irrespective of the presence of glucose (i, Fig. 4*B*) (37). As a control, we also constructed the *ywlE-lacZ* strain with Pspac-*mntH* and observed similarly elevated *ywlE-lacZ* expression (ii, Fig. 4*B*). Moreover, construction of a triple mutant *mntH mntA ycsG* was failed in several trials, while the triple mutant was obtained when the strain carried *amyE*::Pxyl-*ycsG*. This triple mutant showed retarded growth in MC medium under the condition of leaky *ycsG* expression without xylose addition (Fig. S2*B*). These results supported the nature of *ycsG*, that is, the third Mn^2+^ importer in *B. subtilis*.

We also tested the *yknUV* genes encoding an ABC transporter without substrate-binding protein, which has been annotated as an exporter with unknown substrates (38, 39). In the *yknV* disruptant, the GI of *ywlE-lacZ* was abolished (iii, Fig. 4*B*). To perform a complementation test, we constructed a *ywlE-lacZ* strain with the *yknV* disruption and ectopic *yknV* transcribed by the xylose-inducible promoter Pxyl (40). Ectopic and artificial expression of *yknV* resulted in the original GI phenotype (iv, Fig. 4*B*), indicating that *yknV* is responsible for the GI of *ywlE-lacZ* expression. It should be noted that GI was observed even in the absence of xylose, suggesting that trace activity of Pxyl is sufficient for YknV activity in the cell.

### Functional characterization of *ycsG* encoding Mn^2+^ importer and *yknUV* encoding Mn^2+^ exporter

Next, the cellular Mn^2+^ concentrations in the mutants were measured to determine the nature of the putative transporters (Fig. 4*D*). In Pspac-*ycsG, ycsG* and downstream genes, including *ycsI*, are simultaneously upregulated by the Pspac promoter (Fig. 4*E*). In contrast, in Pspac-*ycsI*, only genes downstream of *ycsG* are upregulated by the Pspac promoter (Fig. 4*E*). Mn^2+^ concentrations were enhanced irrespective of glucose in Pspac-*ycsG*, whereas Mn^2+^ concentrations similar to those in the WT were observed with or without glucose in Pspac-*ycsI*(Fig. 4*D*). These show that the effect of Pspac-*ycsG* was not due to the enhancement of downstream genes such as *ycsI*. Hence, we concluded that *ycsG* encodes the Mn^2+^ importer. In the control strain with Pspac-*mntH*, Mn^2+^ concentrations were enhanced, and glucose addition further increased the Mn^2+^ concentrations. In this strain, *mntH* expression was driven by Pspac and its own promoters (Fig. 4*E*). Irrespective of glucose, in the *yknU* and *yknV* disruptants, Mn^2+^ concentrations were elevated, whereas they were not changed in the *yknX* disruptant, ruling out the possible polar effect of *yknUV* disruption on *yknX*. Thus, we concluded that YknUV was involved in Mn^2+^ export. To date, however, the ABC transporter for Mn^2+^ export is unidentified (17).

### Expression of *ycsG*

**Figure 5:**
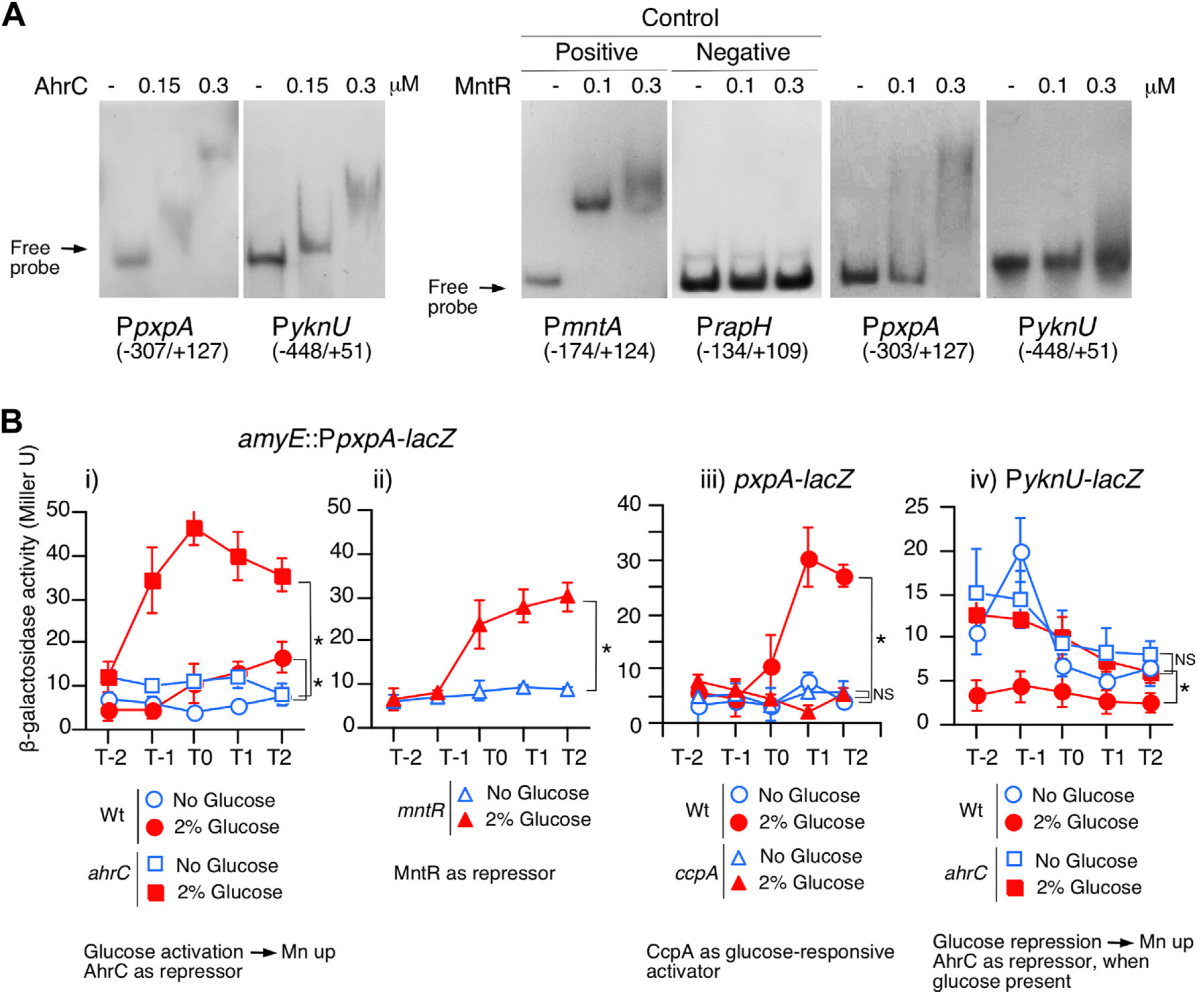
**AhrC/MntR-binding and expression of two newly identified Mn**^**2+**^**transporter loci.** (*A*) EMSA. Concentrations of AhrC/MntR and probe names are shown. *Numbers in parentheses* show nucleotides position to the relative to the translation start point for *yknU* and *rapH*. For *mntA* and *pxpA*, position to the relative to the transcription start point. (*B*) β-Galactosidase activities. CPRG was used for P*yknU-lacZ*. 2-Nitrophenyl-β-D-Galactopyranoside was used for the others. Significant differences in the glucose effect in each strain at T2 were determined using nonpaired *t* test. ∗*p* < 0.05; NS, no significant differences. The *x*-axis represents the growth time in hours relative to the end of vegetative growth (T0). Cells were grown in sporulation medium with (*closed symbols*) or without (*open symbols*) 2% glucose and sampled hourly. Strains: P*ycsF-lacZ* (wt, OAM1027; *ahrC*, OAM1028), *ycsF-lacZ* (wt, YCSFd; *ccpA*, OAM1031), and P*yknU-lacZ* (wt, OAM1025; *ahrC*, OAM1026). CPRG, chlorophenolred β-D-galactopyranoside.

To examine the direct binding of AhrC to P*pxpA* driving *ycsG* (importer), we performed EMSA using protein concentrations for specific binding. P*pxpA* was bound by AhrC, demonstrating direct regulation of P*pxpA* by AhrC (Fig. 5*A*). Next, we performed EMSA using MntR, as enhancement of *ycsG* transcripts was observed in RNA-Seq for MntR (Fig. 4*A*). MntR directly bound to P*pxpA* at concentrations of MntR within those permitting specific binding for P*mntA* (Fig. 5*A*). Next, we analyzed P*pxpA* expression. For the P*pxpA-lacZ* fusion, glucose activated its expression, leading to an increase in Mn^2+^ concentration, and further *ahrC* disruption increased the elevated expression, contrary to the RNA-Seq results due to unknown reason (i, Fig. 5*B*). P*pxpA*-*lacZ* contains the 5′-UTR of *pxpA*, which may work for post-transcription regulation. These results showed that AhrC functions as a repressor irrespective of the presence of glucose. The disruption of *ahrC* did not abolish the GI of the fusion, indicating that the GI of the fusion is not through AhrC. MntR functions as a repressor, as expected, and the GI of the fusion was still observed, indicating that the GI of the fusion is not through MntR (ii, Fig. 5*B*). Thus, we searched for several transcription factors related to this glucose effects and found that CcpA is responsible for the GI of the *ycsG*-containing operon, because GI was abolished in the *ccpA* disruptant (iii, Fig. 5*B*).

### Determination of *cis*-acting sequences of multiple transcription factors in P*pxpA*

**Figure 6:**
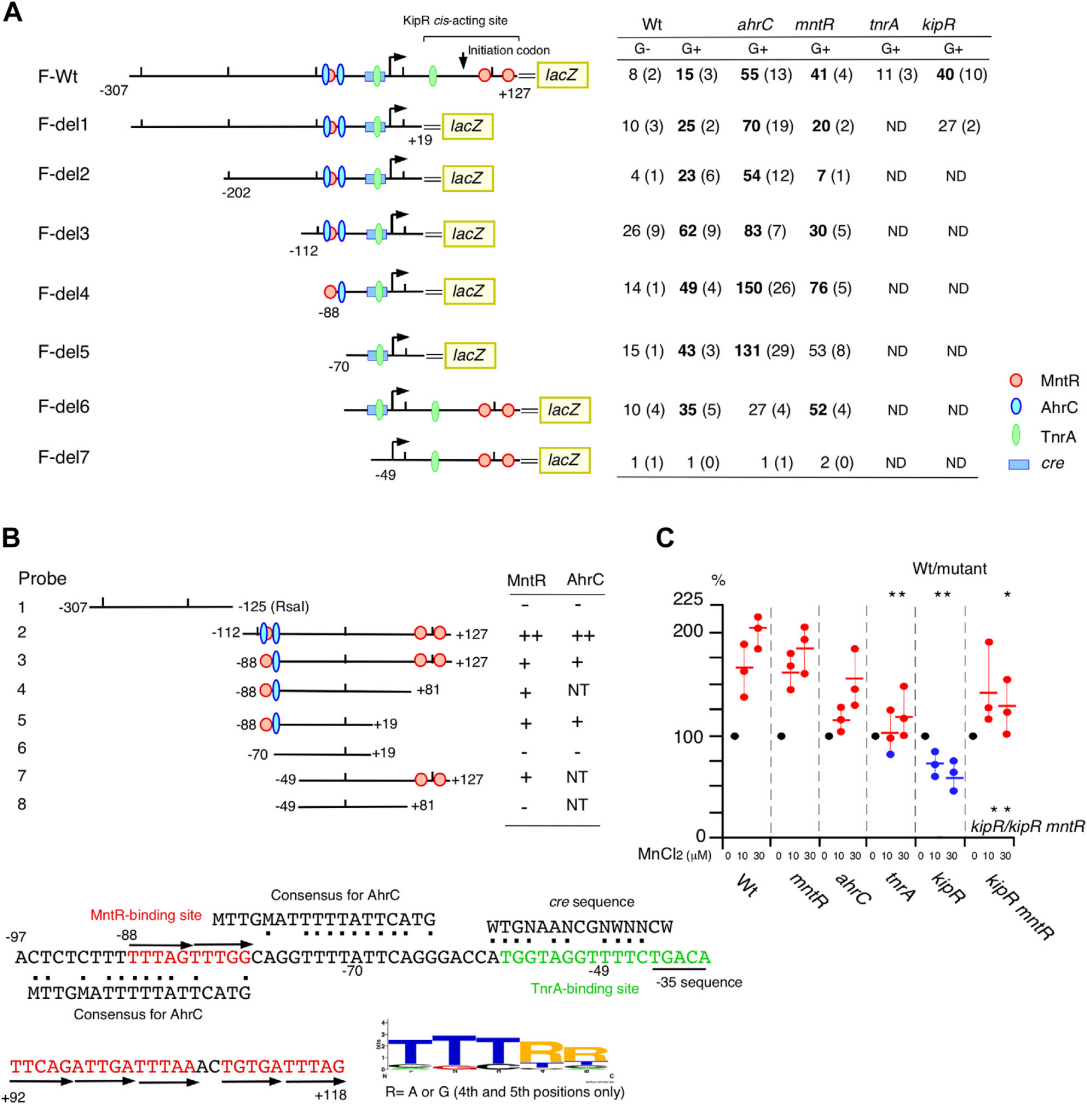
**Expression of P*pxpA-lacZ*.** (*A*) Deletion analysis. Strains were grown in sporulation medium with or without 2% glucose and sampled hourly. Means of peak values (Miller units) from three independent experiments and the SDs are shown in *parenthesis*. Numbers in *bold letter* indicate statistically significant differences (Comparison between with or without glucose in Wt or between wild and disruptant; *p* < 0.05). *Bent arrow* and *double line* show promoter and vector sequence, respectively. Protein stoichiometry in the figure is not taken into account. AhrC consensus is from DBTBS (66). *Numbers along the line* indicate position relative to the transcription start site (SubtiWiki) (67). Strains: F-Wt, OAM1027 (wt); OAM1028 (*ahrC*); OAM1029 (*mntR*); OAM1089 (*tnrA*); OAM1090 (*kipR*); F-del1, OAM1091 (wt); OAM1092 (*ahrC*); OAM1093 (*mntR*); OAM1094 (*kipR*); F-del2, OAM1095 (wt); OAM1096 (*ahrC*); OAM1097 (*mntR*); F-del3, OAM1098 (Wt); OAM1099 (*ahrC*); OAM1100 (*mntR*); F-del4, OAM1101 (Wt); OAM1102 (*ahrC*); OAM1103 (mntR); F-del5, OAM1104 (wt); OAM1105 (*ahrC*); OAM1106 (*mntR*); F-del6, OAM1107 (Wt); OAM1108 (*ahrC*); OAM1109 (*mntR*); F-del7, OAM1110 (wt); OAM1111 (*ahrC*); OAM1112 (*mntR*). (*B*) EMSA results and sequence alignments. *Numbers at the ends of the line* indicate position relative to the transcription start site. ++ indicates the situation where free probe disappeared at low protein levels (0.1 μM for MntR and 0.15 μM for AhrC). EMSA images are shown in Fig. S5*A*. The consensus for MntR binding is generated from all motifs in *pxpA, mntH*, and *mneP*. (*C*) Mn^2+^ response of the P*pxpA-lacZ*. Strains were grown in sporulation medium with 2% glucose and without supplementation of MnCl2. Indicated MnCl2 (final concentrations) was added. For each experiment three independent trials were performed and *asterisks* show *p* < 0.05. The *short horizontal lines* show means of the shown data points. Strains: OAM1027 (wt); OAM1029 (*mntR*); OAM1028 (*ahrC*); OAM1089 (*tnrA*); OAM1090 (*kipR*); and OAM1121 (*kipR mntR*). ND, not determined; NT, not tested.

To obtain deeper understanding of regulation of P*pxpA*, we analyzed the expression of variously deleted promoter-*lacZ* fusions in disruptants of the gene encoding transcription factor. First, we examined CcpA-dependent GI of the constructed fusions, and GI was observed in all the fusions except for F-del7 (Fig. 6*A*). Thus, it is reasonable that CcpA-binding *cre* sequence was detected within the −70/−49 region (Fig. 6) (2). The decrease and increase of P*pxpA* activity in the *tnrA* and *kipR* disruptants, respectively, have been previously reported (36) and we confirmed the expected changes using F-Wt (Fig. 6*A*). Two TnrA-binding sequences were reported (41) and shown in Figure 6*A*, and the upstream site for TnrA is overlapped to the detected *cre* sequence. AhrC-binding sequences were conserved in many bacteria (32) and we detected two adjacent candidates in P*pxpA*. In EMSA, DNA probe 2 up to −112 position showed high affinity to AhrC, while DNA probe 3 up to −88 position showed low affinity to AhrC (see 0.15 μg of AhrC lanes and smear band in probe 5, Fig. S5*A*). This observation is consistent with the presence of two active AhrC-binding sites. Increases in fusions expression (Wt, del1, del2, del3, and del4) by the *ahrC* disruption supported the EMSA results. However, increased expression of del5 in the *ahrC* disruptant seems to be strange because this fusion does not carry any AhrC-binding site. Contrary to this, the del6 fusion expression up to −70 position did not change in the *ahrC* disruptant, which is consistent with the EMSA results. RNA-seq of the *ahrC* disruptant revealed significantly increased *tnrA* expression (Table S1), which may have promoted del5 expression. If so, introducing *tnrA* disruption in the del5 fusion strain with *ahrC* would suppress del5 expression. Our experiment confirmed that this was the case (data not shown). As introducing *tnrA* disruption to F-Wt with *ahrC* resulted in still 2.5-fold enhanced expression in OAM1123 (Table S2), del5 expression in the *ahrC* disruptant may be due to the artificial deletion of the fusion structure. Next, deletion of the downstream region from +1 resulted in the loss of *mntR*- and *kipR*-dependent promoter repression (F-del1). This suggested MntR binding to this region and indeed binding to the +81/+127 region was observed (Fig. 6*B*). The three fusions expression (del1, 2, and 3) was decreased in the *mntR* disruptant, suggesting the positive role of MntR in the upstream region from +1 and additional binding of MntR to the region. EMSA revealed the MntR binding to the −88/−70 region. The expression of del4 with this region increased in the *mntR* disruptant, showing in this fusion MntR plays a negative role contrary to the above three fusions. The apparent contradiction is resolved by considering that the upstream bound AhrC is absent in this fusion, so the anti-AhrC activity of the MntR is lost. Indeed, putative MntR *cis*-acting site (direct repeat of TTTRG) is within the upstream AhrC-binding site, thus MntR would function as an anti-repressor through competition for binding to this sequence. However, it should be noted that overall MntR-regulation is apparently repressive. In the *ahrC mntR* double disruptant slight additive enhanced expression of P*pxpA* was observed (Fig. S6). This is consistent with the downstream MntR-binding site being independent of the AhrC-binding sites. The direct and triple repeat of TTTRG is also within the downstream region required for the effect of MntR, suggesting this motif would be MntR-binding motif.

### MntR-binding motif in regulatory region of *mntH* and *mneP*

For *mntH* and *mneP*, minimum MntR-binding regions were determined with multiple binding sites (22, 23). Thus, we examined whether the putative MntR-binding sequences detected in P*pxpA* are in the regulatory regions of *mntH* and *mneP*. The low expression of *mntH-lacZ*-Wt fusion in the WT was 3-fold enhanced by *mntR* disruption. MntR was bound to the Wt probe, generating two bands (Fig. S5*B*). Deleting 37 bases from the 3′-end of Wt resulted in 8-fold enhancement of the fusion expression, and further enhancement was observed in the *mntR* disruptant. In EMSA, using probe del1 MntR generated single band with lower mobility. Deleting further 24 bases resulted in MntR-independent enhanced expression and no MntR binding. These stepwise alterations of fusion expression and shift patterns in EMSA showed that the +67/+31 and +30/+6 regions contain independent MntR-responsive elements. Indeed, within the +6/+67 region three independent TTTRR repeats were detected. Next, the *mneP* regulatory region was analyzed using EMSA. The previous report showed the minimum MntR-binding region in *mneP*, that is, the −100/+133 region (23). We confirmed this in EMSA. The −386/+118 probe was completely shifted by the binding of MntR at 0.1 μM (Fig. S5*C*). Deletion of 41 bases resulted in scarce MntR binding, indicating that this region contains MntR *cis*-acting site(s) and the two direct repeat of the TTTRR motif were detected. These results supported the notion that the TTTRR motif is recognized by MntR. As the both promoters were regulated by AhrC, in addition to *mneS* and *mntA*, we scanned the four promoter sequences and detected putative AhrC-binding sites (Fig. S5). We introduced *ahrC* disruption into the P*mntH-lacZ* strain with the disruption of *mntR* and examined fusion expression. The elevated fusion expression in the *mntR* disruptant decreased in the double mutant, which is consistent with independent location of AhrC- and MntR-binding sites (Fig. S6).

### Induction of P*pxpA* by Mn^2+^

The expression of the genes encoding two Mn^2+^ importers, *mntH* and *mntABCD*, was repressed by under high Mn^2+^ conditions through Mn^2+^-activated MntR (22, 42). We therefore investigated whether the P*pxpA* expression was altered by Mn^2+^ addition. Contrary to the expectation, P*pxpA* was induced by Mn^2+^ addition (Fig. 6*C*). Thus, we examined the P*pxpA* expression in the disruptants of the transcription factors. In the *mntR* and *ahrC* disruptants, P*pxpA* was still induced similarly to the WT, whereas in the *tnrA* and *kipR* disruptants weakened induction and strong repression of the fusion expression, respectively, were observed. We hypothesized that in the *kipR* disruptant, residual Mn^2+^-activated MntR may repress fusion expression. Thus, the *mntR kipR* double disruptant was constructed, and a modest induction perhaps by TnrA was observed. This indicated that in the absence of induction by KipR, repression by MntR is at work. Thus, the induction of P*pxpA* is mainly caused by KipR and to a lesser extent by TnrA. MntR-dependent repression appears to be hidden by the positive effects of KipR and TnrA, and therefore the effect of *mntR* disruption was not observed in the *mntR* disruptant with the normal *kipR*.

### Expression of P*yknU*

Glucose repressed the expression of P*yknU*-*lacZ*, leading to an increase in Mn^2+^ concentration, and further disruption of *ahrC* increased the glucose-repressed expression (iv, Fig. 5*B*). As AhrC-dependent regulation was expected from the EMSA results, where AhrC, but not MntR, bound to P*yknU* (Fig. 5*A*), this is consistent with the increased expression in the *ahrC* disruptant. Moreover, in the *ahrC* disruptant, the fusion expression was not affected by glucose. Therefore, AhrC functions as a repressor only in the presence of glucose.

## Conclusion

**Figure 7:**
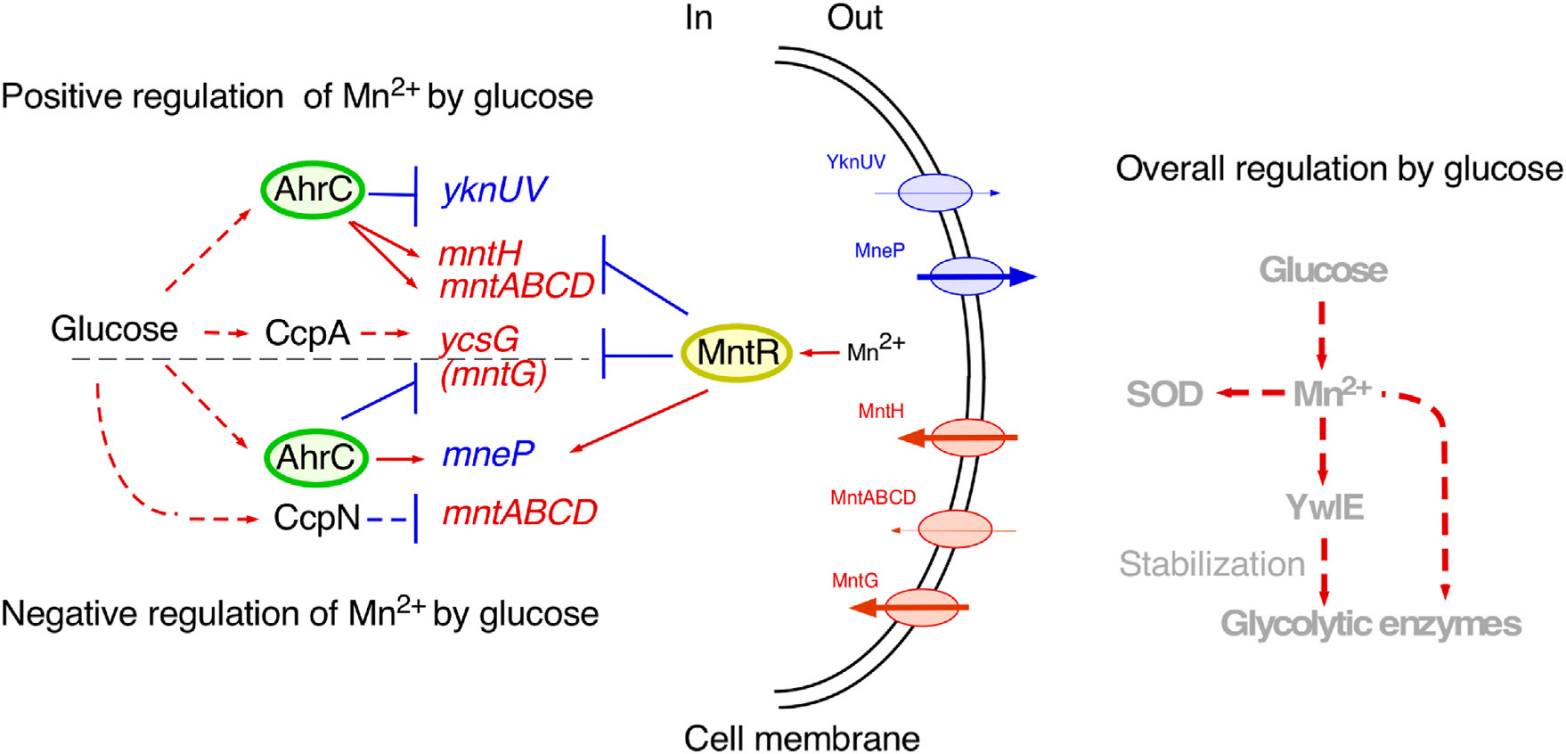
**Schematic representation of glucose-mediated regulation of Mn**^**2+**^**transport.***Left*: *T-bar* and *arrow* indicate inhibition and activation of gene expression, respectively. *Dotted arrow* and *T-bar* indicate indirect effects. *Proteins in red* and *blue* represent Mn^2+^ importer and exporter, respectively. The *arrow width* in the transporter indicates putative overall effects of glucose on transporter genes expression. *Direction of arrows* indicates ion influx or efflux. *Right*: overall glucose-mediated effects of upshift of cellular Mn^2+^ equilibrium is shown. YwlE is a protein arginine phosphatase, which counteracts the arginine phosphorylation of proteins by McsB kinase, leading to protection of the protein from degradation including glycolytic enzymes. Glucose addition results in upshift of central carbon flow including glycolysis, tricarbonic acid cycle, and respiratory chain, leading to generation of toxic reactive oxygen species (ROS). Addition of glucose will increase the demand for superoxide dismutase (SOD).

Based on these analyses, we concluded that two newly identified and known Mn^2+^ transporters contribute to the GI of Mn^2+^ concentrations (Fig. 7). Glucose, both negatively and positively, affects the expression of different genes encoding Mn^2+^ transporters through several transcription factors. However, overall, glucose induces an increase in Mn^2+^ concentrations.

## Discussion

This study shows mechanism of the GI of Mn^2+^ concentrations. An increase in Mn^2+^ concentrations resulted in the induction of *ywlE*. YwlE counteracts arginine phosphorylation of glycolytic enzymes by McsB kinase, thereby protecting the proteins from degradation, which explains the role of GI of *ywlE* (Fig. 7). It was reported that in *S. aureus* cells using glucose as a sole carbon source, the cellular demand for Mn^2+^ and *mntH* expression were increased, compared to cells using amino acids as a sole carbon source, although the mechanisms were not explored (43). Cellular Mn^2+^ concentrations are tightly maintained at steady state levels corresponding to extracellular Mn^2+^ concentrations in the current model (17). Glucose upregulates Mn^2+^ concentrations by imposing AhrC regulation on MntR-regulated *mntH*. The reason for the AhrC-dependent regulation of Mn^2+^ transporter genes is currently unknown; however, we note that the arginase encoded by AhrC-regulated *rocF* requires Mn^2+^ for its enzymatic activity (44). Moreover, glucose addition results in activation of the respiratory chain, generating toxic reactive oxygen species. Superoxide dismutase, which is required for detoxification of reactive oxygen species, also requires Mn^2+^ as a cofactor (45). Since glucose addition increases the demand for superoxide dismutase, GI of Mn^2+^ concentration is advantageous for the cell (Fig. 7).

Mn^2+^ importer YcsG is a member of the natural resistance–associated macrophage protein family of metal ion transporters, which is highly conserved across three kingdoms and also contains MntH (46). YcsG was first identified as a member of the operon-containing *kipI*, which was annotated as a phosphorelay-controlling gene for sporulation initiation (36), and then the three genes in the operon (*ycsF, kipI*, and *kipA*) were reannotated for *pxpABC* encoding ATP-dependent 5-oxoprolinase (34) (Fig. 4*C*). 5-oxoproline is spontaneously generated from glutamine or glutamate and 5-oxoprolinase catalyzes the conversion of 5-oxoproline back into glutamate (34). It is therefore plausible that this operon is regulated by the global nitrogen metabolism regulator TnrA. The arrangement of *pxpABC* and *ycsG* in the same operon is found in *Vibrio fischeri*, *Agrobacterium tumefaciens*, and *Micrococcus luteus* (34). A similar operon structure, in which nitrogen metabolism genes (urea utilization) are associated with *ycsG*, has been reported in *Acinetobacter baumannii* (47). These facts may suggest a link between nitrogen metabolism and Mn^2+^. In previous studies, *ycsG* was reported to be involved in 5-oxoproline utilization; however, this effect did not fully rule out the possible polar effect on downstream *pxpBC* genes (34). This study provides evidence that YcsG is involved in Mn^2+^ import; thus, we renamed this gene as *mntG*. This study revealed that the operon expression is controlled by AhrC, MntR, and CcpA in addition to KipR, which is in this operon, and TnrA (36). Our analysis of their interaction with the promoter region revealed that CcpA and TnrA may compete for binding to the same sequence in P*pxpA*. This situation suggests that CcpA-mediated carbon regulation and TnrA-mediated nitrogen regulation intercrosses at P*pxpA*. The overlapping of the sites for TnrA and CcpA is not unprecedented (48). Characterization of MntR binding to P*pxpA* in addition to *mntH* and *mneP* led to the identification of MntR-recognized sequences and we present a consensus sequence for MntR binding. The likelihood of this consensus will be more certain when the newly identified MntR targets are studied experimentally. The identified sequences do not match the previously reported recognition sequences (23). The discrepancy may be due to the fact that the consensus sequence is partly based on sequences found in the mutational analysis using *lacZ* fusion in the *mneP* regulatory region, where MntR does not actually bind in our study.

P*pxpA* was induced by high Mn^2+^ concentrations. Under high Mn^2+^ conditions, two Mn^2+^ importers MntH and MntABCD were downregulated (22). Thus, at high Mn^2+^ concentrations, changes in the composition of three Mn^2+^ importers occur. The reason for this is still unknown. This Mn^2+^ response of P*pxpA* is mediated by MntR, TnrA, and KipR. The TnrA-dependent Mn^2+^ response has been reported and presented the possible mechanism for this as below (42). TnrA is bound by feedback-inhibited glutamine synthetase by glutamine, resulting in inhibition of TnrA (49). The Mn^2+^ form of glutamine synthetase is more resistant to inhibition by glutamine than the Mg^2+^ form, resulting in less inhibition of TnrA activity (42). *B. subtilis* KipR is poorly characterized; however, the crystal structure of the KipR homologue in *Thermotoga maritima* revealed zinc binding (50). Possible metal binding of *B. subtilis* KipR may be related to Mn^2+^-dependent operon regulation by KipR.

AhrC, which controls arginine metabolic genes expression, is known to be glucose-induced through CcpN *via* ncRNA, SR1 (26, 27). This study expanded the inventory of the genes involved in AhrC-mediated regulation. Thus, the CcpN/AhrC axis appears to be a global glucose regulatory system. In *Streptomyces coelicolor* and *E. coli* ArgR (AhrC analogue) has been shown to regulate numerous genes on a genome-wide scale (51, 52), and in *Enterococcus faecalis*, AhrC regulates genes with functions other than arginine metabolism (53), which corresponds with our RNA-Seq results. L-Arginine is a cofactor that binds to the C-terminal region of AhrC, and thus, arginine would be involved in the AhrC-mediated transcriptional regulation (54).

The MntR-regulated operons also contain many members. The comparative DNA microarray analyses of *mntR* at high Mn^2+^ concentration revealed many genes that belong to the ferric uptake regulator and SigB-regulons (42). A recent proteomic analysis of MntR-regulated proteins found limited numbers of MntR-regulated genes because of the technical limitations of the proteome analysis. They, however, reported the products of genes whose expression fluctuated highly during our RNA-Seq (31).

This study uncovered previously unknown aspects of Mn^2+^ homeostasis control and expansion of the glucose-mediated CcpN/AhrC regulatory axis in many genes. New aspects of Mn^2+^ homeostasis control are important because Mn^2+^ is required for many cellular processes and excess levels of Mn^2+^ can lead to intoxication by this metal.

## Experimental procedures

### Strains, media, plasmid, and β-galactosidase analysis

*B. subtilis* strains and plasmids used in this study are listed in Tables S2. The construction of plasmids is provided in Supplementary methods. A one-step modified competence medium (MC, 100 mM potassium phosphate [pH 7], 3 mM trisodium citrate, 3 mM MgSO_4_, 2% glucose, 22 mg/ml ferric ammonium citrate, 50 mg/ml tryptophan, 0.1% casein lysate, 0.2% potassium glutamate) (30), Schaeffer’s sporulation medium (55), antibiotic III medium (Difco), and lysogeny broth (LB-Lenox) medium (Difco) were used. Antibiotic concentrations were used as described previously (56). Synthetic oligonucleotides were commercially prepared by Tsukuba Oligo Service and are listed in Table S3. Growth conditions and methods for β-galactosidase analysis have been previously described (57). The use of the highly sensitive substrate chlorophenolred β-D-galactopyranoside for the β-galactosidase assay provides 5 to 10 times higher activities than those of 2-Nitrophenyl-β-D-Galactopyranoside, whereas the background activities were at the same level (around 1 Miller units).

### Purification of AhrC and MntR

The *E. coli* strain BL21(DE3) bearing pGEX4T1-ahrC was grown in 600 ml of LB medium (100 μg/ml ampicillin) at 37 °C for 4 h after 1:100 inoculation of overnight culture in LB medium. After 0.5 mM IPTG was added, the cells were further incubated for 20 h at 23 °C. For BL21(DE3) bearing pGEX4T1-mntR, the similar conditions were used, except for the addition of 0.2 mM IPTG and further incubation for 4 h at 23 °C. The cells were harvested, resuspended in 3 ml of the thrombin buffer (20 mM Tris–HCl [pH 8.5], 150 mM NaCl, 2.5 mM CaCl_2_), and disrupted by French pressure cell. After centrifugation (25,000 rpm, 20 min, 4 °C), 2 ml of Glutathione-Sepharose 4B resin slurry (GE Healthcare) was added to the supernatant and gently stirred for 30 min. The mixture was then packed into a column and washed twice with a 10-column volume of the same buffer. After adding biotinylated-thrombin (Novagen) (2U/0.5 ml), the column was left for 20 h at 23 °C. Next, 2 ml of thrombin buffer containing 300 mM NaCl was added to the column. The resulting eluate was passed through a 0.5 ml of Streptavidin-agarose (Novagen) column. After SDS-PAGE analysis, the protein solution was dialyzed against a buffer containing 10% glycerol, 10 mM Tris–HCl [pH 7.5], 1 mM DTT, and 100 mM NaCl, and aliquots of the resultant supernatant were stored at −80 °C after centrifugation. The purified AhrC and MntR proteins that were produced in *E. coli* cells were almost intact but had with two additional amino acids derived from the BamHI restriction site of pGEX4T1 at the N terminus.

### Electromobility shift assays

EMSA was performed using the essentially same methods as the previously published procedures (23). Appropriate amounts of purified AhrC or MntR were added to a final volume of 14 μl buffer containing 5% glycerol, 10 mM Tris–HCl [pH 7.5], 43 mM NaCl, 1 mM MnCl_2_, 1 mM DTT, 1 μg of poly[dI-dC (deoxyinosinic-deoxycytidylic acid)] (Sigma-Aldrich), and biotinylated DNA probe. After adding the protein, the reaction mixture was left for 15 min at 23 °C, following which 2 μl of loading buffer (10% glycerol, 40 mM Tris-acetate buffer [pH 7.5], and 2 mg/ml bromophenol blue) were added and applied to a 5% polyacrylamide gel, and electrophoresis was performed in 40 mM Tris-acetate buffer at 4 °C. The detection of biotin-labeled DNA has been previously described (56).

### Measurement of Mn concentrations

Cells were grown in 50 ml of sporulation medium with or without 2% glucose. Aliquots of 4 ml of the cell culture were harvested at T2. The processes of washing, cell lysis, protein concentration assay, and pretreatment of samples with HNO_3_ were the same as the previously published procedures (22). The 800 μl cleared cell lysate solution was mixed with 3.2 ml of 0.08 M HNO_3_ solution. The Mn concentrations in these solutions were measured using quadrupole inductively coupled plasma mass spectrometry (Agilent 7800, Agilent, Santa Clara). H_2_ gas flow (10 L/min) into the collision cell was used for Mn measurements. ^55^Mn was used as the measurement isotopes and ^115^In was used as an internal standard. The uptake time for each isotope is 0.3 s.

### RNA isolation and RNA-Seq analysis

*B. subtilis* WTe (168), *ahrC* (OAM995) *mntR* (OAM996), and *mneP* (OAM993) strains were newly prepared by transformation of the gene disruption into the WT strain so as to obtain a clean genome background. The cells were grown in 50 ml of sporulation medium with 2% glucose, and 4 ml of cell culture was sampled at T2 for RNA isolation. Four independent cultures were used for each experiment. RNA was isolated from the cells collected by centrifugation using an RNeasy mini kit (Qiagen) with DNase I (Takara) treatment, according to the manufacturer’s instructions. RNA quality was confirmed based on an RNA integrity number >7 using an Agilent RNA 6000 Nano Kit in an Agilent 2100 Bioanalyzer (Agilent Technologies). Ribosomal RNA elimination and complementary DNA library construction was performed using a NEBNext rRNA Depletion Kit (Bacteria) and NEBNext Ultra Ⅱ RNA Library Prep Kit (Illumina) for 1000 ng of total RNA, according to the manufacturer’s protocol. The library was sequenced on the Illumina sequencing platform (Illumina NextSeq 500), and 2 × 75-bp paired-end reads were generated. Adapter sequences in each read were removed using CLC Genomics Workbench 20× software (Qiagen) (https://digitalinsights.qiagen.com/products-overview/discovery-insights-portfolio/analysis-and-visualization/qiagen-clc-genomics-workbench/). The cleaned read data were mapped to the reference genome (RefSeq assembly accession: GCF_000009045.1). Mapping parameters were as follows: mismatch cost, 2; insertion cost, 3; deletion cost, 3; length fraction, 0.8; and similarity fraction, 0.8. DEGs of each condition and control were identified with significant thresholds of a FC ≥ |2|, and FDR adjusted *p*-value (q-value) <0.05 was obtained by a generalized linear model approach using the CLC Genomics Workbench built-in tools differential expression for RNA-Seq.

## Data availability

Original sequence reads were deposited in the DRA/SRA database (accession number: DRR445917-DRR445928).

## Supporting information

This article contains supporting information 4, 7, 13, 22, 23, 30, 35, 37, 58, 59, 60, 61, 62, 63, 64, 65.

## Conflict of interest

The authors declare that they have no conflicts of interest with the contents of this article.
