# Supplementary material for: Glucose controls manganese homeostasis through transcription factors regulating known and newly identified manganese transporter genes in Bacillus subtilis
Source: J Biol Chem. 2023 Jul 17;299(8):105069. doi: 10.1016/j.jbc.2023.105069 (PMC10448178; doi:10.1016/j.jbc.2023.105069)
Supplement: Tables S1–S3 and Figs. S1–S6 [file mmc1.pdf]

Glucose controls manganese homeostasis through transcription factors regulating known and newly-identified manganese transporter genes in *Bacillus subtilis*

Mitsuo Ogura, Minenosuke Matsutani, Kei Asai and Michio Suzuki

Supplementary Methods

Plasmid construction

To construct pUKM504-ywlD and pUKM504-mneS, PCR products were amplified by using the oligonucleotide pairs pUKM-ywlD-F-B/pUKM-ywlD-R-H and pUKM-ydxT-F-B/pUKM-ydxT-R-H, digested with BamHI/HindIII, and cloned into pUKM504 treated with the same enzymes (60). To construct pUKM504-yknV, pUKM504-mntA, and pUKM504-kipR, PCR products were amplified by using the oligonucleotide pairs pUKM-yknV-F-B/pUKM-yknV-R-Sa, pUKM-ytgA-F-B/pUKM-ytgA-R-Sa, and pUKM-kipR-F-B/pUKM-kipR-R-Sa, digested with BamHI/SalI, and cloned into pUKM504 treated with the same enzymes (60). To construct pIS284-ywlDE, pIS284-ywlDE+E, pIS284-argG, pIS284-pyrR, pIS284-mgtE, and pIS284-czcD, the PCR products amplified by using the oligonucleotide pairs pIS-PywlD-F(E)/pIS-PywlD-R(B), pIS-PywlD-F(E)/pMut-PywlE-F(B), pIS-argG-E/pIS-argG-B, pIS-pyrR-F(E)/pIS-pyrR-R(B), pIS-mgtE-E/pIS-mgtE-B, and pIS-czcD-E/pIS-czcD-B, were digested with EcoRI/BamHI and cloned into pIS284 treated with the same enzymes (61). To construct pIS284-ycsF-Wt, pIS284-dhbA, and pIS284-feuA, the PCR products amplified by using the oligonucleotide pairs pIS-ycsF-E/pIS-ycsF-H-bio, pIS-dhbA-E/pIS-dhbA-H, and pIS-feuA-E/pIS-feuA-H were digested with EcoRI/HindIII and cloned into pIS284 treated with the same enzymes (62). The derivatives of pIS-ycsF-Wt were constructed by the similar methods to that described above using different oligonucleotide pairs shown in Table S3. To construct pIS-mntH-Wt, pIS284-argC and pIS-hom, the PCR products amplified by using the oligonucleotide pairs pIS-ydaR-B/pIS-ydaR-H, pIS-argC-B/pIS-argC-H and pIS-hom-B/pIS-hom-H, were digested with BamHI/HindIII and cloned into pIS284 treated with the same enzymes (62). The derivatives of pIS-mntH-Wt were constructed by the similar methods to that described above using different oligonucleotide pairs shown in Table S3. To construct pMutin-PmntA, pMutin-PmneP, pMutin-PmneS, and pMutin-PyknU, PCR products were amplified by

using the oligonucleotide pairs pIS-ytgA-E/pIS-ytgA-B, pMut-ydfM-E/pMut-ydfM-B, pMut-ydxt-E/pMut-ydxt-B, and pIS-yknU-E/pMut-PyknU-B digested with EcoRI/HindIII, and cloned into pMUTIN3 treated with the same enzymes (37). To construct pMutin-PmntH, PCR products amplified by using the oligonucleotide pair pMut-ydaR-H/pMut-ydaR-B were digested with BamHI/HindIII and cloned into pMUTIN3 treated with the same enzymes (37). To construct pMUTIN-His-mntH, PCR products were amplified by using the oligonucleotide pair pMut-His-ydaR-H/pMut-His-ydaR-E, digested with HindIII/EcoRI, and cloned into pMUTIN-His treated with the same enzymes (63). To construct pMUTIN-His-ycsF, PCR products were amplified by using the oligonucleotide pair PMut-His-ycsF-F-E/PMut-His-ycsF-R-Xh, digested with EcoRI/XhoI, and cloned into pMUTIN-His treated with the same enzymes (63). To construct pMUTIN-His-ycsG, PCR products were amplified by using the oligonucleotide pair PMut-His-ycsG-F-H/PMut-His-ycsG-R-Xh, digested with HindIII/XhoI, and cloned into pMUTIN-His treated with the same enzymes (63). To construct pX-yknV and pX-ycsG, PCR products were amplified by using the oligonucleotide pairs pX-yknV-Spe/pX-yknV-Bam and pX-ycsG-Spe/pX-ycsg-Bam, digested with BamHI/SpeI, and cloned into pX treated with the same enzymes (40). To construct pGEX-4T1-ahrC and pGEX-4T1-mntR, PCR products were amplified by using the oligonucleotide pairs ahrC-His-B/ahrC-chitin-R (Xh) and pGEX-mntR-B/mntR-chitin-R (Xh), digested with BamHI/XhoI, and cloned into pGEX-4T1 treated with the same enzymes (Qiagen, Hilden, Germany).

Strain construction

The *ycsG*::Sp^r unit in OAM1032 was constructed using PCR. Briefly Sp^r from pDG1729 (64) and the upstream and downstream regions of *ycsG* with overlapping regions to Sp^r were amplified using primers listed in Table S4 and then combined by PCR. The unit was transformed into *B. subtilis* 168. Total DNA was taken from the resultant Sp^r strain for PCR-based confirmation of the expected chromosomal structure.

Table S1 (Excel file). Differentially-expressed genes in *ahrC* and *mntR* strains. Red (FDR, <0.05, $\log_2$[FC], 1), Green (FDR, <0.001, $\log_2$[FC], 1), Blue (FDR, <0.001, $\log_2$[FC], 2).

Table S2. Strains and plasmids used in the study.

Strain	Genotype	Reference or source
168	*trpC2*	Laboratory stock
OAM992	*trpC2 mntA* (Km^r) *mntH* (Em^r, *lacZ*::Tc^r)	This study
OAM993	*trpC2 mneP* (Em^r, *lacZ*::Tc^r)	This study
OAM994	*trpC2 mneS* (Km^r)	This study
TF4	*trpC2 ahrC* (Cm^r)	65
OAM995	*trpC2 ahrC* (Cm^r::Km^r)	This study
OAM996	*trpC2 mntR* (Em^r)	This study
OAM997	*trpC2 ywlD* (Km^r)	This study
TF10	*trpC2 ccpA* (Cm^r)	65
YQZBd	*trpC2 ccpN* (Em^r)	66
OAM998	*trpC2 ccpN* (Em^r, *lacZ*::Tc^r)	This study
YKNUd	*trpC2 yknU* (*lacZ*, Pspac-*yknV*, Em^r)	61
OAM999	*trpC2 yknV* (Km^r)	This study
YKNXd	*trpC2 yknX* (*lacZ*, Pspac-*yknY*, Em^r)	66
OAM1032	*trpC2 ycsG* (Sp^r)	This study
OAM1088	*trpC2 kipR* (Km^r)	This study
OAM1033	*trpC2 amyE*::P*xyl-ycsG, xylR* (Cm^r)	This study
OAM1034	*trpC2 ycsG* (Cm^r) *amyE*::P*xyl-ycsG, xylR* (Cm^r)	This study
OAM1035	*trpC2 ycsG* (Sp^r) *mntH* (Em^r, *lacZ*::Tc^r) *mntA* (Km^r) *amyE*::P*xyl-ycsG, xylR* (Cm^r)	This study
OAM1000	*trpC2* Pspac-*mntH* (Em^r)	This study
OAM1001	*trpC2* Pspac-*ycsG* (Em^r)	This study
OAM1002	*trpC2* Pspac-*ycsI* (Em^r)	This study
OAM888	*trpC2* P*ywlE-lacZ* (Tc^r)	7
OAM1003	*trpC2* P*ywlE-lacZ* (Tc^r) *mntA* (Km^r) *mntH* (Em^r, *lacZ*::Tc^r)	This study
OAM1004	*trpC2* P*ywlE-lacZ* (Tc^r) *mneP* (Em^r, *lacZ*::Tc^r)	This study
OAM1006	*trpC2* P*ywlE-lacZ* (Tc^r) *ahrC* (Cm^r::Km^r)	This study
OAM1007	*trpC2* P*ywlE-lacZ* (Tc^r) *mntR* (Em^r)	This study
OAM1008	*trpC2* P*ywlE-lacZ* (Tc^r) *ywlD* (Km^r)	This study
OAM1009	*trpC2* P*ywlE-lacZ* (Tc^r) Pspac-*mntH* (Em^r)	This study
OAM1010	*trpC2* P*ywlE-lacZ* (Tc^r) Pspac-*ycsG* (Em^r)	This study
OAM1011	*trpC2* P*ywlE-lacZ* (Tc^r) *yknV* (Km^r)	This study
OAM1012	*trpC2* P*ywlE-lacZ* (Tc^r) *yknV* (Km^r) *amyE*::P*xyl-yknV, xylR* (Cm^r)	This study
OAM1013	*trpC2 amyE*::P*ywlDE-lacZ* (Cm^r)	This study
OAM1082	*trpC2 amyE*::[P*ywlDE*+P*ywlE*]-*lacZ* (Cm^r)	This study
OAM1014	*trpC2* P*mntA*::pMut-PmntA (Em^r)	This study
OAM1015	*trpC2* P*mntH*::pMut-PmntH (Em^r)	This study
OAM1016	*trpC2* P*mneP*::pMut-PmneP (Em^r)	This study
OAM1017	*trpC2* P*mneS*::pMut-PmneS (Em^r)	This study
OAM1018	*trpC2* P*mntA*::pMut-PmntA (Em^r) *ahrC* (Cm^r::Km^r)	This study
OAM1019	*trpC2* P*mntH*::pMut-PmntH (Em^r) *ahrC* (Cm^r::Km^r)	This study
OAM1020	*trpC2* P*mneP*::pMut-PmneP (Em^r) *ahrC* (Cm^r::Km^r)	This study
OAM1021	*trpC2* P*mneS*::pMut-PmneS (Em^r) *ahrC* (Cm^r::Km^r)	This study
OAM1022	*trpC2* P*mntH*::pMut-PmntH (Em^r::Tc^r) *mntR* (Em^r)	This study
OAM1023	*trpC2* P*mntH*::pMut-PmntH (Em^r::Tc^r) *ahrC* (Cm^r::Km^r) *mntR* (Em^r)	This study
OAM1024	*trpC2* P*mntA*::pMut-pmntA (Em^r) *ccpN* (Em^r, *lacZ*::Tc^r)	This study
OAM1025	*trpC2* P*yknU*::pMut-PyknU (Em^r)	This study
OAM1026	*trpC2* P*yknU*::pMut-PyknU (Em^r) *ahrC* (Cm^r::Km^r)	This study
OAM1027	*trpC2 amyE*::P*ycsF-lacZ* (Cm^r) (-307/+127[1])	This study
OAM1028	*trpC2 amyE*::P*ycsF-lacZ* (Cm^r) (-307/+127[1]) *ahrC* (Cm^r::Km^r)	This study
OAM1029	*trpC2 amyE*::P*ycsF-lacZ* (Cm^r) (-307/+127[1]) *mntR* (Em^r)	This study
OAM1030	*trpC2 amyE*::P*ycsF-lacZ* (Cm^r) (-307/+127[1]) *ahrC* (Cm^r::Km^r) *mntR* (Em^r)	This study
OAM1089	*trpC2 amyE*::P*ycsF-lacZ* (Cm^r) (-307/+127[1]) *tnrA* (Cm^r::Km^r)	This study
OAM1090	*trpC2 amyE*::P*ycsF-lacZ* (Cm^r) (-307/+127[1]) *kipR* (Km^r)	This study
OAM1121	*trpC2 amyE*::P*ycsF-lacZ* (Cm^r::Tc^r) (-307/+127[1]) *kipR* (Km^r) *mntR* (Em^r)	This study
OAM1122	*trpC2 amyE*::P*ycsF-lacZ* (Cm^r::Tc^r) (-307/+127[1]) *ahrC* (Cm^r::Km^r)	This study
OAM1123	*trpC2 amyE*::P*ycsF-lacZ* (Cm^r::Tc^r) (-307/+127[1]) *ahrC* (Cm^r::Km^r) *tnrA* (Cm^r)	This study
OAM1091	*trpC2 amyE*::P*ycsF-lacZ* (Cm^r) (-307/+19[1])	This study
OAM1092	*trpC2 amyE*::P*ycsF-lacZ* (Cm^r) (-307/+19[1]) *ahrC* (Cm^r::Km^r)	This study
OAM1093	*trpC2 amyE*::P*ycsF-lacZ* (Cm^r) (-307/+19[1]) *mntR* (Em^r)	This study
OAM1094	*trpC2 amyE*::P*ycsF-lacZ* (Cm^r) (-307/+19[1]) *kipR* (Km^r)	This study
OAM1095	*trpC2 amyE*::P*ycsF-lacZ* (Cm^r) (-202/+19[1])	This study
OAM1096	*trpC2 amyE*::P*ycsF-lacZ* (Cm^r) (-202/+19[1]) *ahrC* (Cm^r::Km^r)	This study
OAM1097	*trpC2 amyE*::P*ycsF-lacZ* (Cm^r) (-202/+19[1]) *mntR* (Em^r)	This study
OAM1098	*trpC2 amyE*::P*ycsF-lacZ* (Cm^r) (-112/+19[1])	This study
OAM1099	*trpC2 amyE*::P*ycsF-lacZ* (Cm^r) (-112/+19[1]) *ahrC* (Cm^r::Km^r)	This study
OAM1100	*trpC2 amyE*::P*ycsF-lacZ* (Cm^r) (-112/+19[1]) *mntR* (Em^r)	This study
OAM1101	*trpC2 amyE*::P*ycsF-lacZ* (Cm^r) (-88/+19[1])	This study
OAM1102	*trpC2 amyE*::P*ycsF-lacZ* (Cm^r) (-88/+19[1]) *ahrC* (Cm^r::Km^r)	This study

OAM1103	*trpC2 amyE*::P*ycsF-lacZ* (Cm^r) (-88/+19^1) *mntR* (Em^r)	This study
OAM1104	*trpC2 amyE*::P*ycsF-lacZ* (Cm^r) (-70/+19^1)	This study
OAM1105	*trpC2 amyE*::P*ycsF-lacZ* (Cm^r) (-70/+19^1) *ahrC* (Cm^r::Km^r)	This study
OAM1106	*trpC2 amyE*::P*ycsF-lacZ* (Cm^r) (-70/+19^1) *mntR* (Em^r)	This study
OAM1119	*trpC2 amyE*::P*ycsF-lacZ* (Cm^r::Tc^r) (-70/+19^1) *ahrC* (Cm^r::Km^r)	This study
OAM1120	*trpC2 amyE*::P*ycsF-lacZ* (Cm^r::Tc^r) (-70/+19^1) *ahrC* (Cm^r::Km^r) *mntR* (Em^r)	This study
OAM1107	*trpC2 amyE*::P*ycsF-lacZ* (Cm^r) (-70/+127^1)	This study
OAM1108	*trpC2 amyE*::P*ycsF-lacZ* (Cm^r) (-70/+127^1) *ahrC* (Cm^r::Km^r)	This study
OAM1109	*trpC2 amyE*::P*ycsF-lacZ* (Cm^r) (-70/+127^1) *mntR* (Em^r)	This study
OAM1110	*trpC2 amyE*::P*ycsF-lacZ* (Cm^r) (-49/+127^1)	This study
OAM1111	*trpC2 amyE*::P*ycsF-lacZ* (Cm^r) (-49/+127^1) *ahrC* (Cm^r::Km^r)	This study
OAM1112	*trpC2 amyE*::P*ycsF-lacZ* (Cm^r) (-49/+127^1) *mntR* (Em^r)	This study
YCSFd	*trpC2* P*ycsF-lacZ* (Em^r)	66
OAM1031	*trpC2* P*ycsF-lacZ* (Em^r) *ccpA* (Cm^r)	This study
OAM1113	*trpC2 amyE*::P*mntH-lacZ* (Cm^r) (-245/+67^1)	This study
OAM1114	*trpC2 amyE*::P*mntH-lacZ* (Cm^r) (-245/+67^1) *mntR* (Em^r)	This study
OAM1115	*trpC2 amyE*::P*mntH-lacZ* (Cm^r) (-245/+30^1)	This study
OAM1116	*trpC2 amyE*::P*mntH-lacZ* (Cm^r) (-245/+30^1) *mntR* (Em^r)	This study
OAM1117	*trpC2 amyE*::P*mntH-lacZ* (Cm^r) (-245/+6^1)	This study
OAM1118	*trpC2 amyE*::P*mntH-lacZ* (Cm^r) (-245/+6^1) *mntR* (Em^r)	This study
YUAAd	*trpC2* P*ktrA-lacZ* (Em^r)	66
OAM1078	*trpC2* P*ktrA-lacZ* (Em^r) *ahrC* (Km^r)	This study
OAM825	*trpC2 amyE*::P*pyrR-lacZ* (Cm^r)	4
OAM1036	*trpC2 amyE*::P*pyrR-lacZ* (Cm^r) *mntR* (Em^r)	This study
OAM1037	*trpC2 amyE*::P*argC-lacZ* (Cm^r)	This study
OAM1038	*trpC2 amyE*::P*argC-lacZ* (Cm^r) *mntR* (Em^r)	This study
OAM1039	*trpC2 amyE*::P*argG-lacZ* (Cm^r)	This study
OAM1040	*trpC2 amyE*::P*argG-lacZ* (Cm^r) *mntR* (Em^r)	This study
OAM1073	*trpC2 amyE*::P*czcD-lacZ* (Cm^r)	This study
OAM1074	*trpC2 amyE*::P*czcD-lacZ* (Cm^r) *mntR* (Em^r)	This study
OAM1075	*trpC2 amyE*::P*mgtE-lacZ* (Cm^r)	This study
OAM1076	*trpC2 amyE*::P*mgtE-lacZ* (Cm^r) *mntR* (Em^r)	This study
OAM1045	*trpC2* P*pucR-lacZ* (Em^r::Tc^r)	Derivative of BSF2277 (66)
OAM1046	*trpC2* P*pucR-lacZ* (Em^r::Tc^r) *ahrC* (Km^r)	This study
OAM1047	*trpC2* P*pucR-lacZ* (Em^r::Tc^r) *mntR* (Em^r)	This study
OAM1048	*trpC2* P*pucA-lacZ* (Em^r::Tc^r)	Derivative of BSF2309 (66)
OAM1049	*trpC2* P*pucA-lacZ* (Em^r::Tc^r) *ahrC* (Km^r)	This study
OAM1050	*trpC2* P*pucA-lacZ* (Em^r::Tc^r) *mntR* (Em^r)	This study
OAM1051	*trpC2* P*pucG-lacZ* (Em^r::Tc^r)	Derivative of BSF2285 (66)
OAM1052	*trpC2* P*pucG-lacZ* (Em^r::Tc^r) *ahrC* (Km^r)	This study
OAM1053	*trpC2* P*pucG-lacZ* (Em^r::Tc^r) *mntR* (Em^r)	This study
OAM914	*trpC2 amyE*::P*frlB-lacZ* (Cm^r)	13
OAM1063	*trpC2 amyE*::P*frlB-lacZ* (Cm^r) *ahrC* (Em^r)	This study
OAM1064	*trpC2 amyE*::P*frlB-lacZ* (Cm^r) *mntR* (Km^r)	This study
OAM1060	*trpC2* P*artP-lacZ* (Em^r::Tc^r)	Derivative of YQIXd (66)
OAM1061	*trpC2* P*artP-lacZ* (Em^r::Tc^r) *ahrC* (Km^r)	This study
OAM1062	*trpC2* P*artP-lacZ* (Em^r::Tc^r) *mntR* (Em^r)	This study
OAM1054	*trpC2 amyE*::P*spoIIE-lacZ* (Cm^r)	Constructed by Sato T
OAM1055	*trpC2 amyE*::P*spoIIE-lacZ* (Cm^r) *ahrC* (Km^r)	This study
OAM1056	*trpC2 amyE*::P*spoIIE-lacZ* (Cm^r) *mntR* (Em^r)	This study
OAM1057	*trpC2* P*spoIIAA-lacZ* (Em^r::Tc^r)	Derivative of the strain constructed by Sato T
OAM1058	*trpC2* P*spoIIAA-lacZ* (Em^r::Tc^r) *ahrC* (Km^r)	This study
OAM1059	*trpC2* P*spoIIAA-lacZ* (Em^r::Tc^r) *mntR* (Em^r)	This study
OAM1043	*trpC2* P*skfA-lacZ* (Em^r::Tc^r)	Derivative of YBCOd (66)
OAM1087	*trpC2* P*skfA-lacZ* (Em^r::Tc^r) *ahrC* (Km^r)	This study
OAM1044	*trpC2* P*skfA-lacZ* (Em^r::Tc^r) *mntR* (Em^r)	This study
OAM1079	*trpC2* P*kimA-lacZ* (Em^r::Tc^r)	Derivative of YDAOd (66)
OAM1080	*trpC2* P*kimA-lacZ* (Em^r::Tc^r) *ahrC* (Km^r)	This study
OAM1081	*trpC2* P*kimA-lacZ* (Em^r::Tc^r) *mntR* (Em^r)	This study
OAM1083	*trpC2 amyE*::P*hom-lacZ* (Cm^r)	This study
OAM1084	*trpC2 amyE*::P*hom-lacZ* (Cm^r) *ahrC* (Km^r)	This study
OAM1085	*trpC2 amyE*::P*hom-lacZ* (Cm^r) *mntR* (Em^r)	This study
OAM1041	*trpC2 amyE*::P*dhbA-lacZ* (Cm^r)	This study
OAM1086	*trpC2 amyE*::P*dhbA-lacZ* (Cm^r) *ahrC* (Km^r)	This study
OAM1042	*trpC2 amyE*::P*dhbA-lacZ* (Cm^r) *mntR* (Em^r)	This study
OAM1065	*trpC2 amyE*::P*feuA-lacZ* (Cm^r)	This study
OAM1066	*trpC2 amyE*::P*feuA-lacZ* (Cm^r) *ahrC* (Em^r)	This study
OAM1067	*trpC2 amyE*::P*feuA-lacZ* (Cm^r) *mntR* (Km^r)	This study
OAM838	*trpC2 amyE*::P*hisZ-lacZ* (Cm^r)	4
OAM1068	*trpC2 amyE*::P*hisZ-lacZ* (Cm^r) *ahrC* (Em^r)	This study

OAM1069	*trpC2 amyE*::P*hisZ-lacZ* (Cm^r) *mntR* (Km^r)	This study
OAM1070	*trpC2* P*pftA-lacZ* (Em^r::Tc^r)	Derivative of OAM950 (35)
OAM1071	*trpC2* P*pftA-lacZ* (Em^r::Tc^r) *ahrC* (Km^r)	This study
OAM1072	*trpC2* P*pftA-lacZ* (Em^r::Tc^r) *mntR* (Em^r)	This study

Plasmid	Description	Reference or source
pUKM504	pUC19 bearing Km^r	56
pUKM504-ywlD	pUKM504 carrying a part of *ywlD*	This study
pUKM504-yknV	pUKM504 carrying a part of *yknV*	This study
pUKM504-mntA	pUKM504 carrying a part of *mntA*	This study
pUKM504-mneS	pUKM504 carrying a part of *mneS*	This study
pUKM504-kipR	pUKM504 carrying a part of *kipR*	This study
pIS284	Amp^r *amyE*::*lacZ* Cm^r	65, I Smith
pIS284-ywlD	Amp^r *amyE*::P*ywlDE-lacZ* Cm^r	This study
pIS284-[ywlDE+E]	Amp^r *amyE*::[P*ywlDE*+*PywlE*]-*lacZ* Cm^r	This study
pIS284-ycsF-Wt	Amp^r *amyE*::P*ycsF-lacZ* Cm^r (-307/+127[1])	This study
pIS284-ycsF-del1	Amp^r *amyE*::P*ycsF-lacZ* Cm^r (-307/+19[1])	This study
pIS284-ycsF-del2	Amp^r *amyE*::P*ycsF-lacZ* Cm^r (-202/+19[1])	This study
pIS284-ycsF-del3	Amp^r *amyE*::P*ycsF-lacZ* Cm^r (-112/+19[1])	This study
pIS284-ycsF-del4	Amp^r *amyE*::P*ycsF-lacZ* Cm^r (-88/+19[1])	This study
pIS284-ycsF-del5	Amp^r *amyE*::P*ycsF-lacZ* Cm^r (-70/+19[1])	This study
pIS284-ycsF-del6	Amp^r *amyE*::P*ycsF-lacZ* Cm^r (-70/+127[1])	This study
pIS284-ycsF-del7	Amp^r *amyE*::P*ycsF-lacZ* Cm^r (-49/+127[1])	This study
pIS284-mntH-Wt	Amp^r *amyE*::P*mntH-lacZ* Cm^r (-245/+67[1])	This study
pIS284-mntH-del1	Amp^r *amyE*::P*mntH-lacZ* Cm^r (-245/+30[1])	This study
pIS284-mntH-del2	Amp^r *amyE*::P*mntH-lacZ* Cm^r (-245/+6[1])	This study
pIS284-pyrR	Amp^r *amyE*::P*pyrR-lacZ* Cm^r	This study
pIS284-argG	Amp^r *amyE*::P*argG-lacZ* Cm^r	This study
pIS284-argC	Amp^r *amyE*::P*argC-lacZ* Cm^r	This study
pIS284-czcD	Amp^r *amyE*::P*czcD-lacZ* Cm^r	This study
pIS284-mgtE	Amp^r *amyE*::P*mgtE-lacZ* Cm^r	This study
pIS284-hom	Amp^r *amyE*::P*hom-lacZ* Cm^r	This study
pIS284-dhbA	Amp^r *amyE*::P*dhbA-lacZ* Cm^r	This study
pIS284-feuA	Amp^r *amyE*::P*feuA-lacZ* Cm^r	This study
pMutinIII	Insertion vecter, Amp^r, Em^r, *lacZI*	37
pMutin-PmntA	pMutinIII carrying P*mntA*	This study
pMutin-PmntH	pMutinIII carrying P*mntH*	This study
pMutin-PmneP	pMutinIII carrying P*mneP*	This study
pMutin-PmneS	pMutinIII carrying P*mneS*	This study
pMutin-PyknU	pMutinIII carrying P*yknU*	This study
pMUTIN-His	Amp^r Em^r His-tag Pspac	63
pMUTIN-His-mntH	Amp^r Em^r C-terminal region of *mntH* His-tag Pspac	This study
pMUTIN-His-ycsF	Amp^r Em^r C-terminal region of *ycsF* His-tag Pspac	This study
pMUTIN-His-ycsG	Amp^r Em^r C-terminal region of *ycsG* His-tag Pspac	This study
pX	Amp^r *amyE*::*xylR*-Pxyl Cm^r	40
pX-yknV	Amp^r *amyE*::*xylR*-P*xyl-yknU* Cm^r	This study
pX-ycsG	Amp^r *amyE*::*xylR*-P*xyl-ycsG* Cm^r	This study
pLacZ::Tc	Amp^r *lacZ*::Tc^r	62
pCm::Km	Amp^r Cm^r::Km^r	67
pCm::Tc	Amp^r Cm^r::Tc^r	67
pEm::Tc	Amp^r Em^r::Tc^r	67
pGEX-4T1	Amp^r GST	Qiagen
pGEX-4T1-mntR	pGEX4T-1 carrying *mntR*	This study
pGEX-4T1-ahrC	pGEX4T-1 carrying *ahrC*	This study

* Relative to the transcription start site

Table S3. Oligonucleotides used for this study.

Name	Sequence (5'-3')	Use/products
pUKM-ywlD-F-B	AAAGGATCCTTGGAATGGGCATGGT	pUKM504-ywlD
pUKM-ywlD-R-H	ATGAAGCTTGTTTTCCGACCAGCAGG	pUKM504-ywlD
pUKM-yknV-F-B	AAGGATCCAATGTGTTAAGAATCAGGTG	pUKM504-yknV
pUKM-yknV-R-Sa	TCAGTCGACAGCCGTCCCAATCGC	pUKM504-yknV
pUKM-ytgA-F-B	AAAGGATCCGCACCTTTACAAAGC	pUKM504-mntA
pUKM-ytgA-R-Sa	TCAGTCGACTCATTCCCAAAGTACGCA	pUKM504-mntA
pUKM-ydxT-F-B	AAAGGATCCTGACGAAGATCATCCATA	pUKM504-mneS
pUKM-ydxT-R-H	ATGAAGCTT TATGTCGAATCCATCTGTC	pUKM504-mneS
pUKM-kipR-F-B	AAAGGATCCTAACCTTAAGTGAACTGGTG	pUKM504-kipR
pUKM-kipR-R-Sa	TCAGTCGACTCGGTTTGTTTGATGTATGC	pUKM504-kipR
pIS-PywlD-F(E)	TCAGAATTCGCCGACAGCGCCTCTTG	pIS284-ywlD, pIS284-[ywlDE+E]
pIS-PywlD-R(B)	TCAGGATCCACATAAATAACCCCCTTGGACAC	pIS284-ywlD
pMut-PywlE-F(B)	AGGGATCCGTCAGTCACCCCTTATTTTTCTC	pIS284-[ywlDE+E]
pIS-ycsF-E	ATCGAATTCGGCTTTACAATGGAGAATG	EMSA, pIS284-ycsF-Wt, -del1
pIS-ycsF-H-bio	biotin-CCGAAGCTTTCTCCTAAATC	EMSA, pIS284-ycsF-Wt, del6, del7, EMSA (del2, del3, del7)
pIS-ycsF-H2	CGGAAGCTTCCGAATTTTTGAACTTTATTTCA	pIS284-ycsF-del1, del2, del3, del4, del5, EMSA (del5)
pIS-ycsF-E2	ATCGAATTCATCGTAAAAGAAACGGCTG	pIS284-ycsF-del2
pIS-ycsF-E3	ATCGAATTCAGAGTCCTAAGATGGACTC	pIS284-ycsF-del3, EMSA (del2)
pIS-ycsF-E3aa	ATCGAATTCTTTAGTTTGGCAGGTTTTATTCT	pIS284-ycsF-del4, EMSA (del3)
pIS-ycsF-E3a	ATCGAATTCTGGGACCATGGTAGG	pIS284-ycsF-del5, del6, EMSA (del6)
pIS-ycsF-E3b	ATCGAATTCTGACAAAAGTATTTCACAAACTG	pIS284-ycsF-del7
pIS-ydaR-B	ATCGGATCCCGGCCATCATCGGGG	pIS284-mntH-Wt
pIS-ydaR-H	ATGAAGCTTGTCTCACCTGAATTCTGTTT	pIS284-mntH-Wt, EMSA
pIS-mntH-H3	ATGAAGCTTCAAAGAGTTTCCTTAAGGCA	pIS284-mntH-del1, EMSA
pIS-mntH-H2	ATGAAGCTTTTATCTACATCATATGACCATCC	pIS284-mntH-del2, EMSA
pIS-ytgA-E	ATCGAATTCACACAGCATGGTTAAGAAA	pMutin-pmntA
pIS-ytgA-B	ATGGGATCCATTTCTCCTCCTCTTTGCCATC	pMutin-pmntA, EMSA
pMut-ydaR-H	ATCAAGCTTCGGCTCTATGATTGCCG	pMutin-pmntH
pMut-ydaR-B	ATCGGATCCTTGTTCATCATGTCTCAC	pMutin-pmntH
pMut-ydfM-E	CTCGAATTCGAGATATTATGTTA	pMutin-pmneP, EMSA
pMut-ydfM-B	ATGGGATCCTCCTATAAAACTGCACAAAAAAA	pMutin-pmneP, EMSA
pMut-ydxt-E	CTCGAATTCCGATTACACCGGCAATC	pMutin-pmneS
pMut-ydxt-B	ATGGGATCCAAAACCTCCTCTCGCTCA	pMutin-pmneS
pIS-yknU-E	ATCGAATTCTTGAATCTATTGATACGGCTATG	pMutin-pyknU
pMut-PyknU-B	CCGGGATCCATCCAGCACCTTTCCAAC	pMutin-pyknU
pMut-His-ydaR-H	TGAAAGCTTGGATTGGGCACGGA	pMUTIN-His-mntH
pMut-His-ydaR-E	GTCTCACCTGAATTCTGTTT	pMUTIN-His-mntH
PMut-His-ycsF-F-E	CGCGAATTCTTTTCGGTCTTGCTGAGA	pMUTIN-His-ycsF
PMut-His-ycsF-R-Xh	GCGCTCGAGTTATGTTGACCTCTGTTCTGA	pMUTIN-His-ycsF
PMut-His-ycsG-F-H	CGCAAGCTTGCGGTTCTAGGCGTTG	pMUTIN-His-ycsG
PMut-His-ycsG-R-Xh	GCGCTCGAGTCAGCTCCACAATTGAGGAA	pMUTIN-His-ycsG
pX-yknV-Spe	AAACTAGTTGGAAAGGTGCTGGATATG	pX-yknV
pX-yknV-Bam	ATGGGATCCTATCCGACCTCATGCGGC	pX-yknV
pX-ycsG-Spe	AAACTAGTCAACATAAAGGAGGAACAATAG	pX-ycsG
pX-ycsG-Bam	ATGGGATCCTCAGCTCCACAATTGAGGAAG	pX-ycsG
ycsG-FF	TCCGGGCTTGCCGGATTT	OAM1032
ycsG-FR	CCAGTCACGTTACGTGCCCATCAGCAATGACCAGC	OAM1032
ycsG-RF	CTAATTGGTAATCAGAGCGCTCGGAACGCTGCTT	OAM1032
ycsG-RR	CAATATGGATCGGCCCTC	OAM1032
Spc-F	ACGTAACGTGACTGGCAAGA	OAM1032
Spc-R	CTGATTACCAATTAGAATGAAT	OAM1032
pIS-argG-E	ATGGAATTCTGCCGGCGTCATTCAGC	EMSA, pIS284-argG
pIS-argG-B	TTCGGATCCGATAAAAATCCCCTCTCAACCG	pIS284-argG
pIS-argC-B	ATGGGATCCATTATGCTCGGGGGCTTTC	EMSA, pIS284-argC
pIS-argC-H	TTGAAGCTTCCCTTCTCCGCTGGATGAATAA	pIS284-argC
pIS-pyrR-F(E)	ATTGAATTCGAACCCATCAAATTCGTGTTC	EMSA, pIS284-pyrR
pIS-pyrR-R(B)	TTCGGATCCTGTGTGACACCTCACAGTTCAT	pIS284-pyrR
pIS-dhbA-E	ATCGAATTCAGCCGGATGAATGATAATGC	pIS284-dhbA
pIS-dhbA-H	CGGAAGCTTATCATCAATTCCTTTCTTCGCTCT	pIS284-dhbA
pIS-feuA-E	ATCGAATTCACACCTTCAGAACAAGCGA	pIS284-feuA
pIS-feuA-H	CGGAAGCTTCTATAGAGCCTCCTGTTCAA	pIS284-feuA
pIS-czcD-E	ATCGAATTCGATGTGCCTCAACAAGACC	pIS284-czcD, EMSA
pIS-czcD-B	CATGGATCCTCCTTACCTAAAGTTTTTAA	pIS284-czcD
pIS-mgtE-E	ATCGAATTCAGGCTGAATATGTCCCTC	pIS284-mgtE, EMSA
pIS-mgtE-B	CATGGATCCGGGACTCGTACCTCCTC	pIS284-mgtE
pIS-hom-B	ATCGGATCCGCTGTCTTCATTTTCGAAAC	pIS284-hom, EMSA
pIS-hom-H	TTCAAGCTTAAAAACTCCACCTTTCTTTTGATTG	pIS284-hom
mntH-bio-F	biotin-TCCCGGCCATCATCGGG	EMSA
gcp-bio (thiL)	biotin-ACACCCGTTCCCATACCGAACA	EMSA
pDG1729-gcp-B2	ATTGGATCCGTTTACATTAATGGCGGTCCGG	EMSA
RapH-bio	biotin-CAACCTCCGCTTTCAGAATC	EMSA
RapH-F1	CCCTTGCATAAAGGGTTC	EMSA
pMUT-His-PyknU-F-E	CGCGAATTCTTTTATTTGCAGGGCTTG	EMSA
yknU-bio-R	biotin-CACTTTTCTGTACGGCCAAT	EMSA

argC-Bio-R	biotin-CCCTCTTGGTCTTTGTGAAA	EMSA
ydfM-bio-R	biotin-TCCTATAAAACTGCACAAAAAAA	EMSA
mneS-bio-F	biotin-GCGCTTTCAGCGAATGTGTG	EMSA
mneS-R	CAGTTCATCATATCTCTCCATAC	EMSA
mneP-bio-F	bioin-TTCACACAGCATGGTTAAGAAA	EMSA
mneP-bio-R3	biotin-GCAACTTTTCTGCTTATTTGTTC	EMSA
ycsF-H2-bio	biotin-TTCCGAATTTTTGAACTTTATTTCA	EMSA (del6)
ycsF-E3b-bio	biotin-TTCTGACAAAAGTATTTCACAAACTG	EMSA (del7, del8)
ycsF-R2	CCCTCCTGCCAAAGCAAA	EMSA (del1, del4, del8)
ycsF-bio-F	biotin-TCGGCTTTACAATGGAGAATG	EMSA (del1)
ycsF-E3aa-bio	biotin-TTTAGTTTGGCAGGTTTTATTCT	EMSA (del4, del5)
pIS-ytgA-E	ATCGAATTCACACAGCATGGTTAAGAAA	EMSA
ytgA-bio-F	biotin-TTCACACAGCATGGTTAAGAAA	EMSA
pyrR-bio-R	biotin-TGTGTGACACCTCACAGTTCAT	EMSA
argG-bio-F	biotin-CTGCCGGCGTCATTCAGC	EMSA
dhbA-bio-F	biotin-AGCCGGATGAATGATAATGC	EMSA
dhbA-R	ATCATCAATTCCTTTCTTCGCTCT	EMSA
czcD-R-Bio	biotin-TCCTCCTTACCTAAAGTTTTTAA	EMSA
mgtE-R-Bio	biotin-CGGGACTCGTACCTCCTC	EMSA
skfA-F	GTCAATCTATTAGGCATCAGAA	EMSA
skfAR-bio-R	biotin-TCATAAGTAAACCTCCTCTC	EMSA
ktrA-F	GACTCAGCCTTGCGGTTG	EMSA
ktrA-R-bio	biotin-TGTTCATATCTCCCTTAGTGAA	EMSA
pucR-F	CAATCTATCACACCAGGAAA	EMSA
pucR-bio-R	biotin-CATAGCGCATTCTCCTTTTC	EMSA
spoIIE-F	CTTTCACGGCGGTAACACGG	EMSA
spoIIE-bio-R	biotin-GAGTTTTTCCAAAGCTTGTCCC	EMSA
spoIIA-F2	GGCCAAGAGCTTGGCACTCTT	EMSA
spoIIA-R-bio	biotin-CTTGATATGATCGGATAATGAGTG	EMSA
artP-F	GAAGGCCATGAGCCGATG	EMSA
artP-bio-R	biotin-GATCCATTTCCCCCGTATTC	EMSA
hom-bio	biotin-AAAAACTCCACCTTTCTTTTGATTG	EMSA
frlB-F-Eco	CTGGAATTCACCGATCAAACATCACAG	EMSA
frlB-R-bio	biotin-CTCAAATCCTTCACTCCTCG	EMSA
feuA-bio-F	biotin-CACACCTTCAGAACAAGCGA	EMSA
feuA-R	CTATAGAGCCTCCTGTTCAA	EMSA
pIS-hisZ-E	ATTGAATTCGCCTGAAAGAATCATCAGG	EMSA
hisZ-bio-R	biotin-ATCTCTCATGCCGTGCGG	EMSA
pftA-bio-F	biotin-CGGTCAGCGATACACTCG	EMSA
kimA-F	GGTAAGTAAATTCCATTTGTGGAAC	EMSA
kimA-R-bio	biotin-CGATGTCTTCCCCTTTTAATTTCTC	EMSA
pftA-R	GCACTCATTTTCTTCACCTCTTTC	EMSA
pGEX-mntR-B	CCGGGATCCATGACAACACCAAGTATGGA	pGEX4T-1-mntR
mntR-chitin-R (Xh)	TTGCTCGAGTTACTGATTATGATGTTCTGTTTTC	pGEX4T-1-mntR
ahrC-His-B	ATTGGATCCATGAACAAAGGCCAGAGGC	pGEX4T-1-ahrC
ahrC-chitin-R (Xh)	TTGCTCGAGTTACAGCAGTTCAAGGAGCC	pGEX4T-1-ahrC

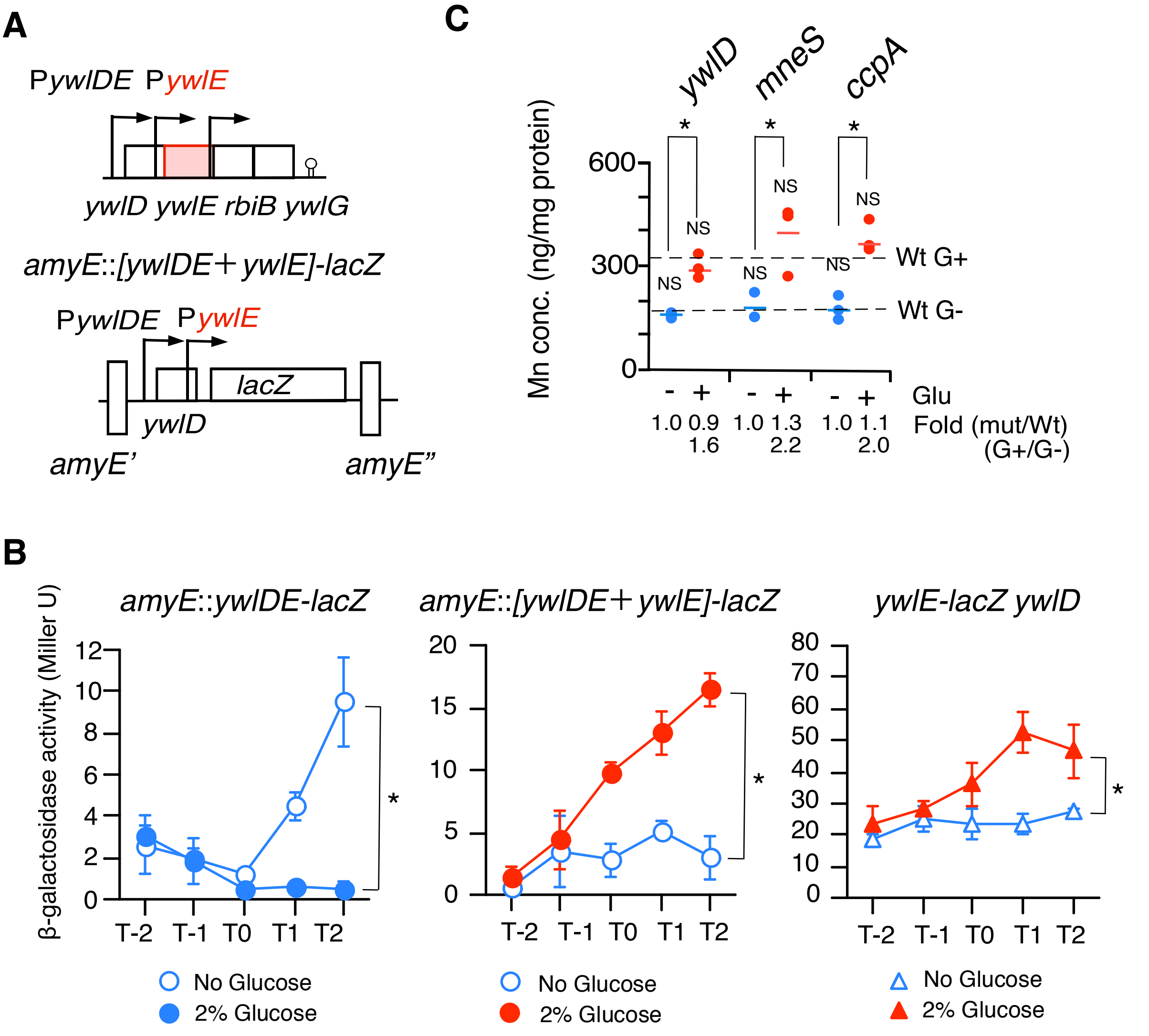

Fig. S1. YwlD is not involved in glucose induction of *ywlE* expression and cellular Mn concentrations. (A) Schematic representations of the region surrounding *ywlE* and the structure of [*ywlDE*+*ywlE*]-*lacZ* at *amyE*. Box, bent arrows, and stem-loop show open reading frame, promoter, and terminator, respectively. **(B)** Expression of *ywlDE-lacZ* (OAM1013), [*ywlDE*+*ywlE*]-*lacZ* (OAM1082) and *ywlE-lacZ* in *ywlD* disruptant (OAM1008). β-Galactosidase activities were shown in Miller units. Means from three independent experiments and the standard deviations are shown. The *x*-axis represents the growth time in h relative to the end of vegetative growth (T0). Cells were grown in sporulation medium with (closed symbols) or without (open symbols) 2% glucose and sampled hourly. Substrate CPRG was used for *ywlE-lacZ* and [*ywlDE*+*ywlE*]-expression. Significant differences in the effects of glucose addition at T2 were determined using nonpaired t-test. * $P < 0.05$. **(C)** Cellular Mn concentrations. T2 cells grown in sporulation medium were harvested and processed. Strains; *ywlD* (OAM997), *mneS* (OAM994), *ccpA* (TF10). "Glu" represents glucose. Three biologically independent samples were measured. Significant differences between Wt and mutants, with or without glucose (* and "NS" above each data point indicate $P < 0.05$ and no significant difference, respectively) and the effect of glucose addition to each strain were determined using nonpaired t- test. * $P < 0.05$; NS, no significant differences. The short horizontal lines show means of the shown data points.

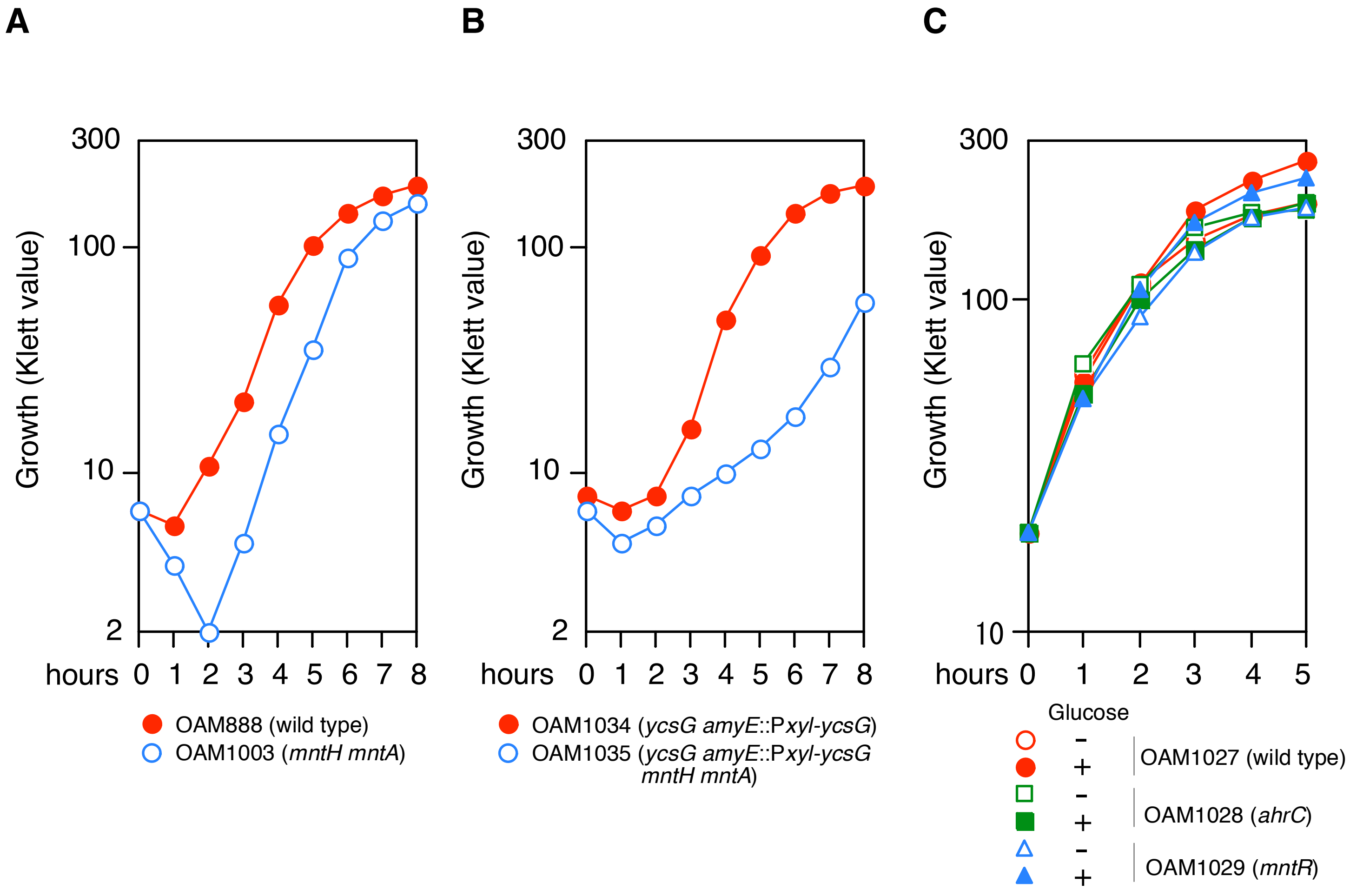

Fig. S2. Cell growth profiles in various mutants. Typical cell growth profiles monitored with a Klett calorimeter (Fisher Scientific, Waltham, MA, USA) are shown. **(A)** and **(B)** Overnight culture grown in LB medium (Difco, MI, USA) was washed with semisynthetic MC medium (100 mM potassium phosphate [pH 7], 3 mM trisodium citrate, 3 mM $MgSO_4$, 2% glucose, 22mg/mL ferric ammonium citrate, 50 mg/mL tryptophan, 0.1% casein lysate, 0.2% potassium glutamate) (30) and inoculated to 4 mL MC medium in an L-tube. **(C)** Cells grown on LB agar plate were inoculated to 50 mL sporulation medium with or without 2% glucose.

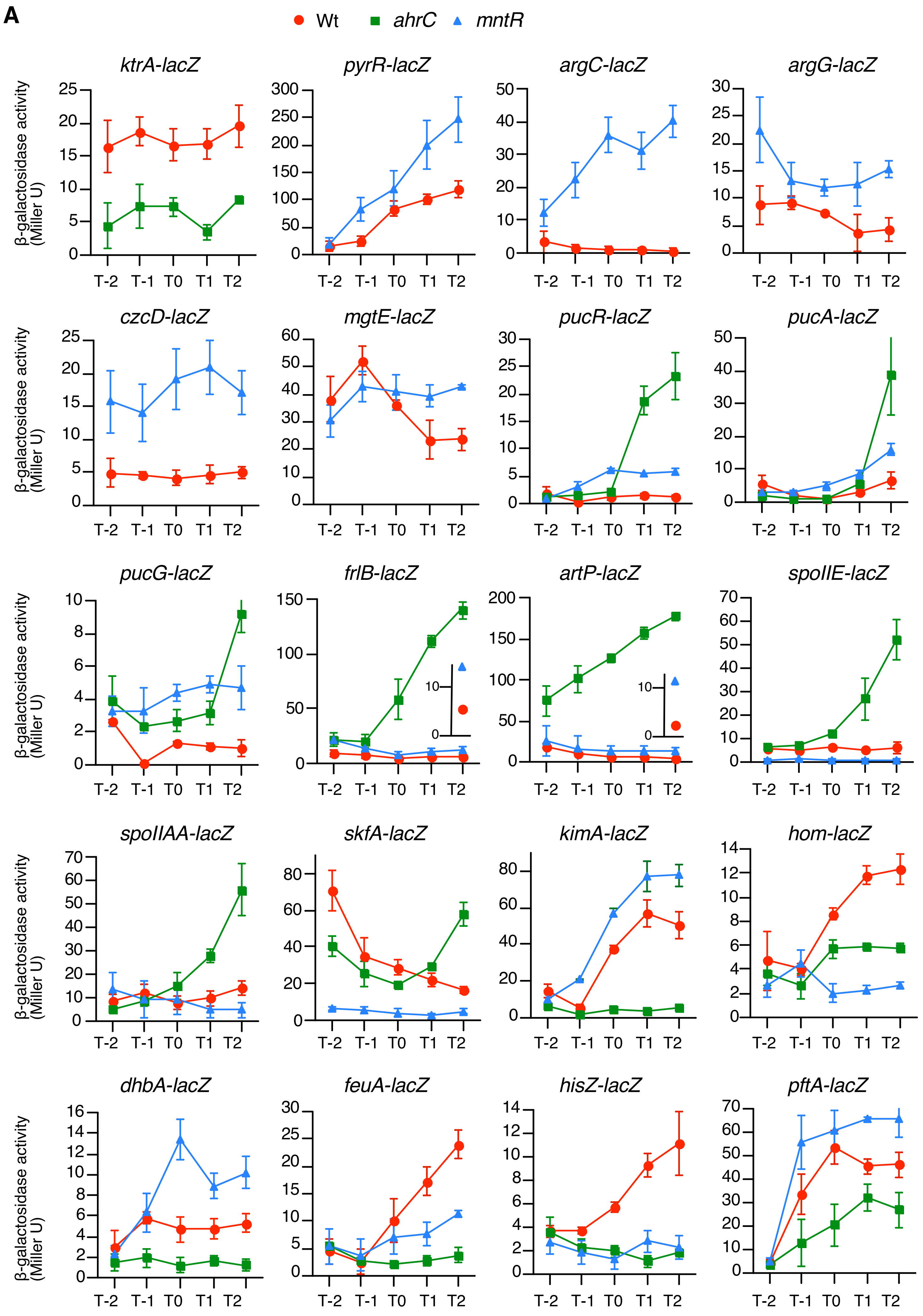
A
Wt
ahrC
mntR
ktrA-lacZ
pyrR-lacZ
argC-lacZ
argG-lacZ
czcD-lacZ
mgtE-lacZ
pucR-lacZ
pucA-lacZ
pucG-lacZ
frlB-lacZ
artP-lacZ
spoIIE-lacZ
spoIIAA-lacZ
skfA-lacZ
kimA-lacZ
hom-lacZ
dhbA-lacZ
feuA-lacZ
hisZ-lacZ
pftA-lacZ
β-galactosidase activity (Miller U)
T-2 T-1 T0 T1 T2

Fig. S3A

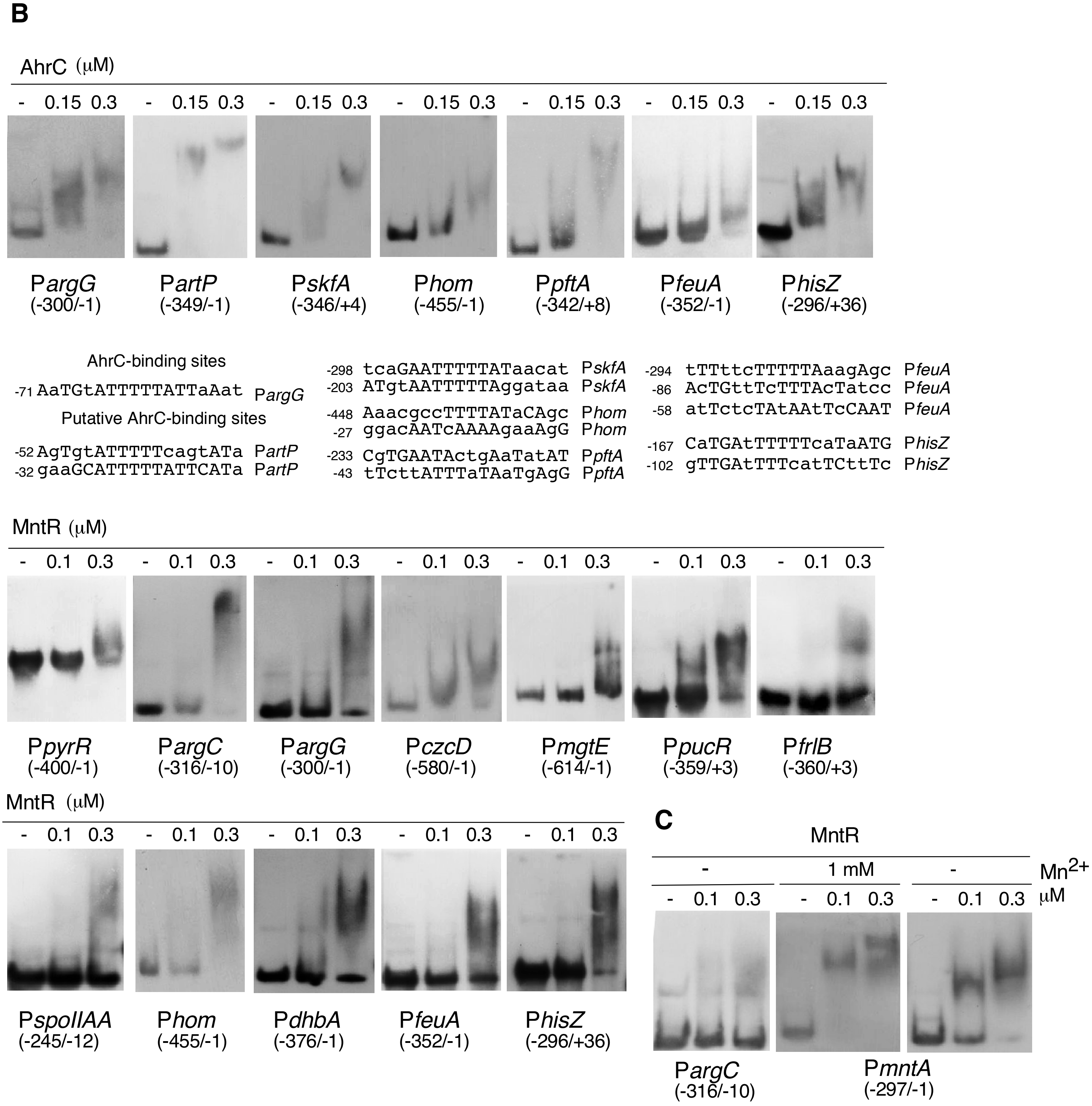

Fig. S3. Expression analysis and AhrC/MntR-binding of the various promoters detected by RNA-Seq. (A) β-Gal analysis. β-Galactosidase activities were show n in Miller units. Means from three independent experiments and the standard deviations are shown. The *x*-axis represents the growth time in h relative to the end of vegetative growth (T0). Cells were grown in sporulation medium with 2% glucose and sampled hourly. Red circles, green squares, and blue triangles show wild type, *ahrC* disruptant, and *mntR* disruptant, respectively. Substrate CPRG was used for *argC-lacZ* and *ktrA-lacZ* expression. The negative effects of the *mntR* disruption on *dhbA* and *kimA* were observed in the RNA-Seq analyses, whereas the positive effects were seen in the β-Gal analysis due to an unknown reason. Since some strains have a strong tendency to develop suppressor mutations after long incubation, the strains were immediately used

after construction of the strains. Strains. *ktrA-lacZ*, YUAAd (Wt), OAM1078 (*ahrC*); *pyrR-lacZ*, OAM825 (Wt), OAM1036 (*mntR*); *argC-lacZ* , OAM1037 (Wt), OAM1038 (*mntR*); *argG-lacZ* , OAM1039 (Wt), OAM1040 (*mntR*); *czcD-lacZ*, OAM1073 (Wt), OAM1074 (*mntR*); *mgtE-lacZ*, OAM1075 (Wt), OAM1076 (*mntR*); *pucR-lacZ*, OAM1045 (Wt), OAM1046 (*ahrC*), OAM1047 (*mntR*); *pucA-lacZ*, OAM1048 (Wt), OAM1049 (*ahrC*), OAM1050 (*mntR*); *pucG-lacZ*, OAM1051 (Wt), OAM1052 (*ahrC*), OAM1053 (*mntR*); *frlB-lacZ*, OAM914 (Wt), OAM1063 (*ahrC*), OAM1064 (*mntR*); *artP-lacZ* , OAM1060 (Wt), OAM1061 (*ahrC*), OAM1062 (*mntR*); *spoIIE-lacZ*, OAM1054 (Wt), OAM1055 (*ahrC*), OAM1056 (*mntR*)); *spoIIAA-lacZ*, OAM1057 (Wt), OAM1058 (*ahrC*), OAM1059 (*mntR*); *skfA-lacZ*, OAM1043 (Wt), OAM1087 (*ahrC*), OAM1044 (*mntR*); *kimA-lacZ*, OAM1079 (Wt), OAM1080 (*ahrC*), OAM1081 (*mntR*); *hom-lacZ*, OAM1083 (Wt), OAM1084 (*ahrC*), OAM1085 (*mntR*); *dhbA-lacZ*, OAM1041 (Wt), OAM1086 (*ahrC*), OAM1042 (*mntR*);*feuA-lacZ*, OAM1065 (Wt), OAM1066 (*ahrC*), OAM1067 (*mntR*); *hisZ-lacZ*, OAM838 (Wt), OAM1068 (*ahrC*), OAM1069 (*mntR*); *pftA-lacZ*, OAM1070 (Wt), OAM1071 (*ahrC*), OAM1072 (*mntR*). Significant differences in the effects of *ahrC* or *mntR* disruption at T2 in the presence of glucose were determined using nonpaired t- test. As all the cases exhibited significant differences ($P < 0.05$), these are not indicated in the Figure. **(B)** EMSA (electromobility shift assay). Concentrations of proteins and probe names are shown. Numbers in parentheses show nucleotides position to the relative to the translation start point. Putative AhrC-binding sites are shown. **(C)** MntR-binding with or without Mn^{2+}.

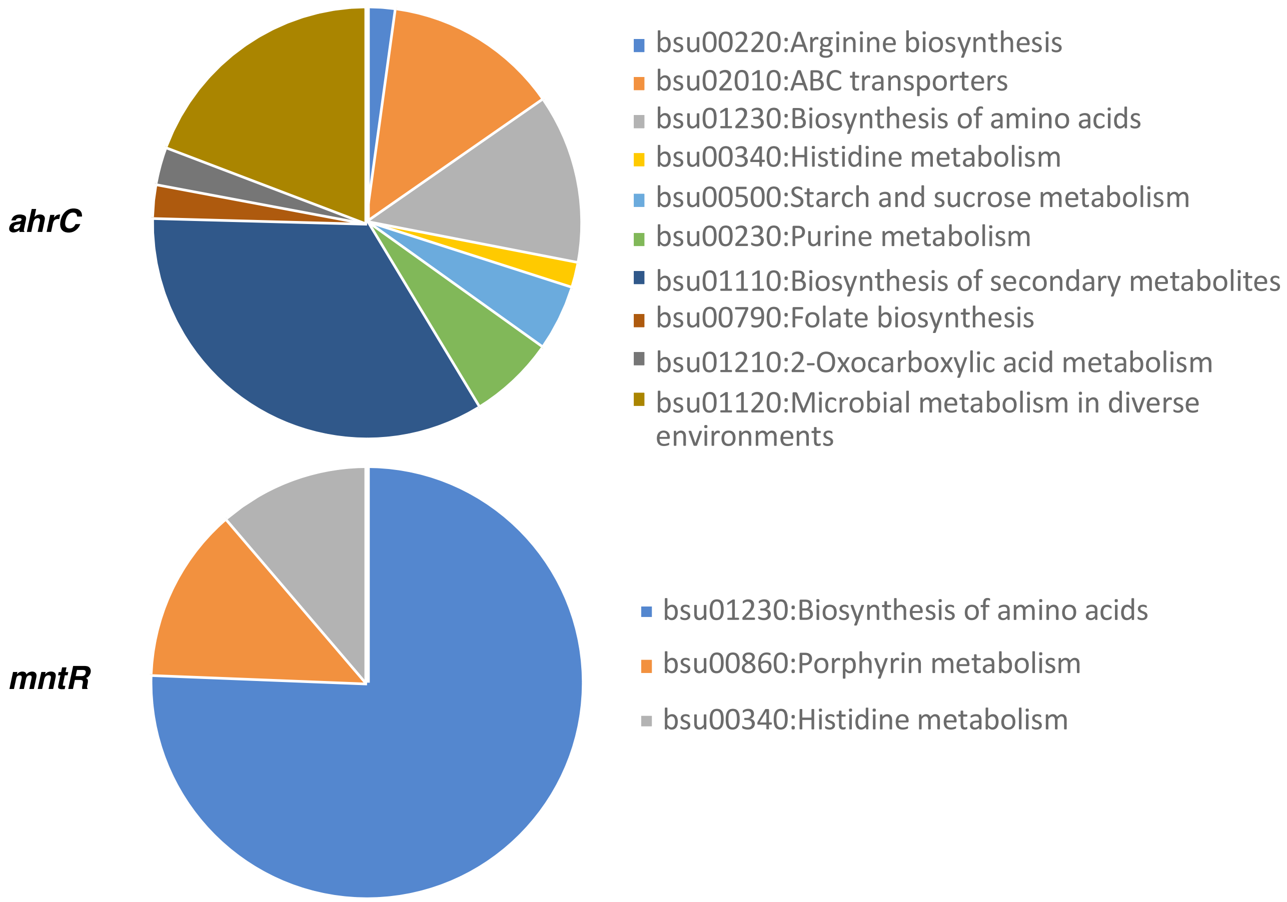

Fig. S4. Enrichment analysis of AhrC for Gene Ontology and KEGG pathway. The analyses were performed using DAVID server (https://david.ncifcrf.gov/). DEGs were used as input for enrichment analysis, and considered as enrichment pathways with significant thresholds of false discovery rate adjusted p-value (FDR) < 0.05.

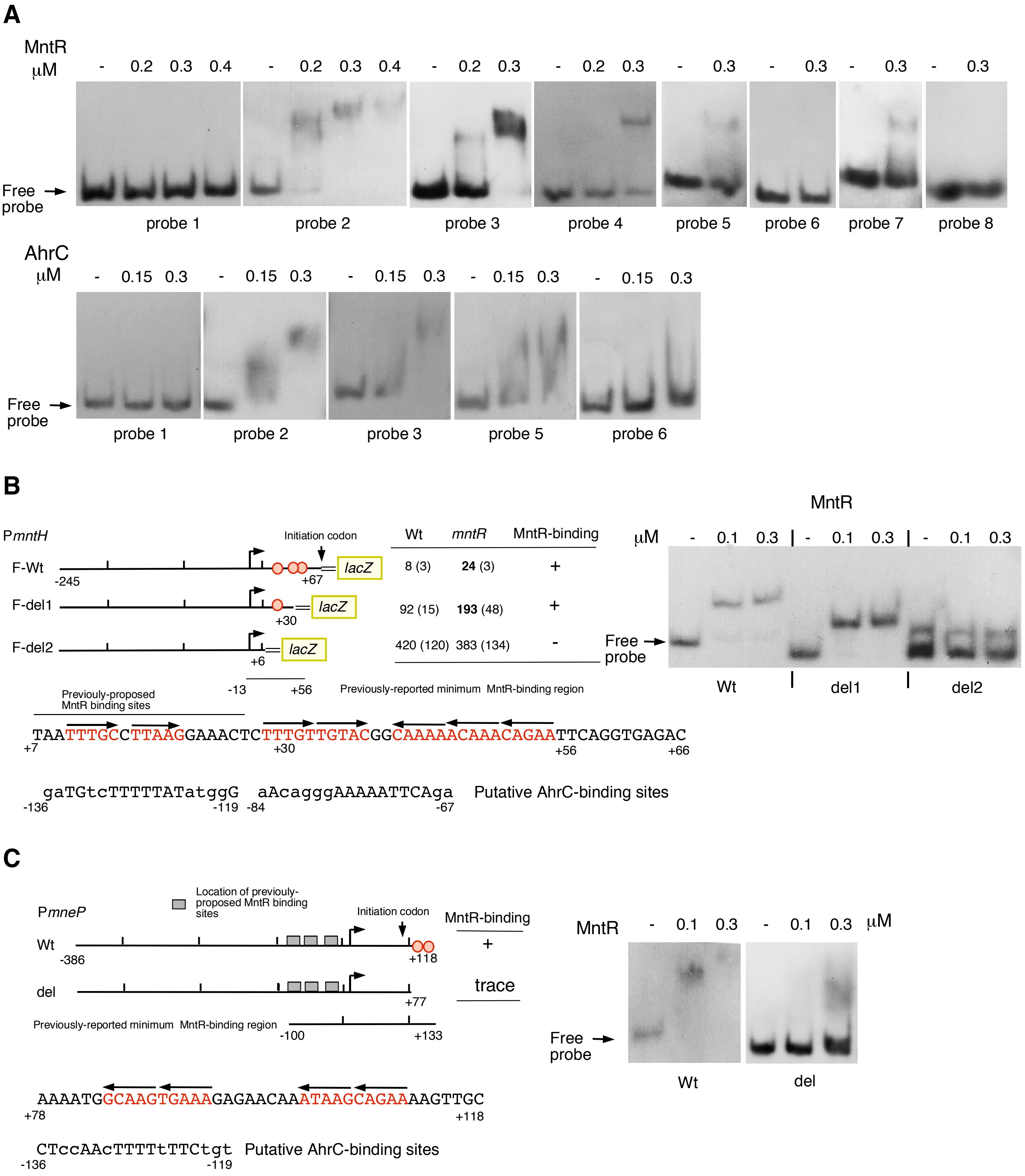

D

Putative AhrC-binding sites in *mneS*

tTgcgtTTTTTATTCgct (-211 to -194)   ATgGgATTTTTggcggaG (-127 to -110)   ggaGcAaAtAAATTggAT (-113 to -96)

Putative AhrC-binding sites in *mntA*

ggTtAAgAAAAAaGCAtT (-169 to -152)   tgTaAATAAAAcaTgctT (+20 to +37)

Fig. S5

Fig. S5. EMSA Results and expression of *mntH-lacZ* (**A**) EMSA images in Fig. 6B. Protein concentrations and probe names are shown. (**B**) and (**C**) Expression analysis of *mntH* and EMSA of *mntH* and *mneP*. Strains were grown in sporulation medium with 2% glucose and sampled hourly. Means of peak values (Miller units) from three independent experiments and the standard deviations are shown in parenthesis. Numbers in bold letter indicate statistically significant differences (Comparison between wild and disruptant; $P < 0.05$). Bent arrow and double line show promoter and vector sequence, respectively. Numbers along the line indicate position relative to the transcription start site (22, 23). The reported minimum MntR binding regions are shown (22, 23). Strains, F-Wt, OAM1113 (wild); OAM1114 (*mntR*); F-del1, OAM1115 (wild); OAM1116 (*mntR*); F-del2, OAM1117 (wild); OAM1118 (*mntR*). Putative MntR-binding sequences are shown in red letters. These sequences for AhrC were within the probe region used in Fig. 2A. (**D**) Putative AhrC-binding sites in *mneS* and *mntA*. Numbers along the line indicate position relative to the transcription start site (22, 23). These sequences were within the probe region used in Fig. 2A.

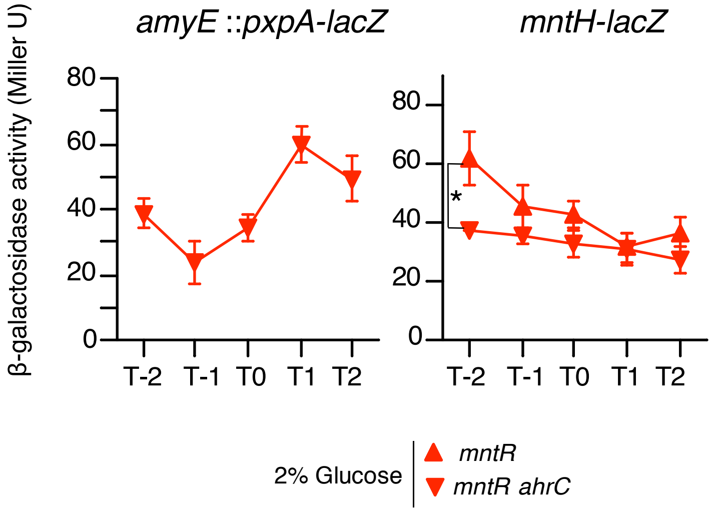

Fig. S6 Expression of P *pxpA* and P *mntH* in *ahrC mntR* disruptant. Strains were grown in sporulation medium with 2% glucose and sampled hourly. Means (Miller units) from three independent experiments and the standard deviations are shown. * P < 0.05). The *x*-axis represents the growth time in h relative to the end of vegetative growth (T0). When the peak values of P*pxpA-lacZ* in the *ahrC* or *mntR* disruptant (Fig. 5B) was compared to that in double disruptant, statistically significant difference was observed (P < 0.05). Strains, OAM1030 (P*pxpA-lacZ, mntR ahrC*), OAM1022 (P*mntH-lacZ, mntR*), OAM1023 (P*mntH-lacZ, mntR ahrC).*
